# Mechanisms of sex determination and X-chromosome dosage compensation

**DOI:** 10.1093/genetics/iyab197

**Published:** 2022-01-06

**Authors:** Barbara J Meyer

**Affiliations:** Department of Molecular and Cell Biology, Howard Hughes Medical Institute, University of California, Berkeley, Berkeley, CA 94720-3204, USA

**Keywords:** sex determination, X-chromosome dosage compensation, X-chromosome counting, *Caenorhabditis elegans*, mRNA splicing regulation, transcriptional regulation, cell fate determination, chromosome structure, histone modification, chromosome segregation, WormBook

## Abstract

Abnormalities in chromosome number have the potential to disrupt the balance of gene expression and thereby decrease organismal fitness and viability. Such abnormalities occur in most solid tumors and also cause severe developmental defects and spontaneous abortions. In contrast to the imbalances in chromosome dose that cause pathologies, the difference in X-chromosome dose used to determine sexual fate across diverse species is well tolerated. Dosage compensation mechanisms have evolved in such species to balance X-chromosome gene expression between the sexes, allowing them to tolerate the difference in X-chromosome dose. This review analyzes the chromosome counting mechanism that tallies X-chromosome number to determine sex (XO male and XX hermaphrodite) in the nematode *Caenorhabditis elegans* and the associated dosage compensation mechanism that balances X-chromosome gene expression between the sexes. Dissecting the molecular mechanisms underlying X-chromosome counting has revealed how small quantitative differences in intracellular signals can be translated into dramatically different fates. Dissecting the process of X-chromosome dosage compensation has revealed the interplay between chromatin modification and chromosome structure in regulating gene expression over vast chromosomal territories.

## Overview

**Figure 1:**
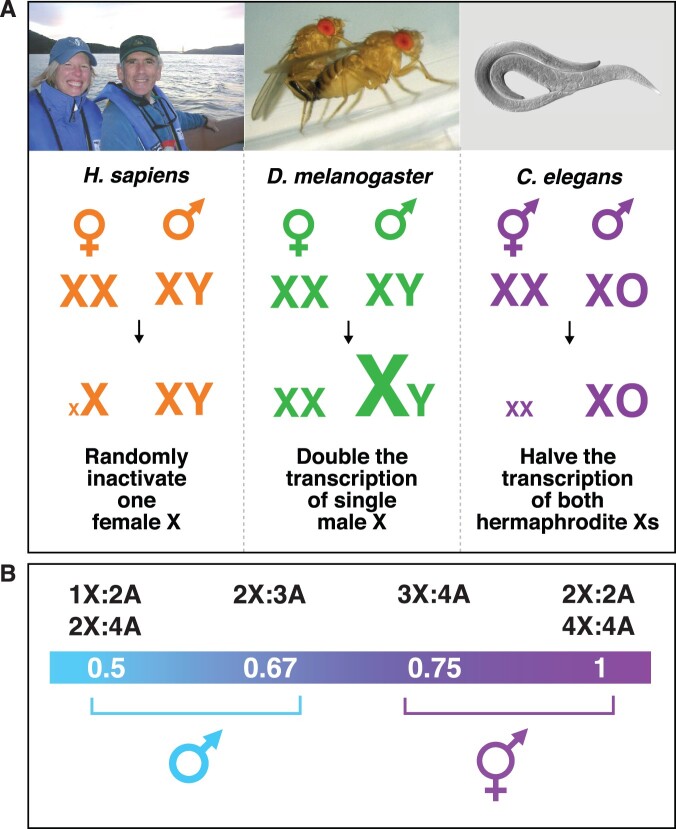
Diverse strategies for X-chromosome dosage compensation. (A) Organisms use different strategies to ensure that males and females or hermaphrodites produce comparable levels of X-linked gene products, despite the twofold difference in X dose between the sexes. Female human and *Mus musculus* mammals (XX) randomly inactivate most genes on one X chromosome. Male *D. melanogaster* fruit flies (XY) double transcription of their singe X chromosome. Hermaphrodite *C. elegans* worms (XX) reduce transcription of both X chromosomes by half. (B) The nematode calculates the ratio of X chromosomes to sets of autosomes to determine sexual fate. Chromosome counting is executed with remarkable precision in the nematode such that diploid animals with one X chromosome (1X:2A, ratio 0.5) and triploid animals with two X chromosomes (2X:3A, ratio of 0.67) become fertile males, while diploid animals with two X chromosomes (2X:2A, ratio 1.0) and tetraploid animals with three X chromosomes (3X:4A, ratio of 0.75) become fertile hermaphrodites. Other organisms like fruit flies discriminate less well such that only a ratio of 0.5 results in fertile males, and a ratio of 1.0 results in fertile females, with intermediate ratios generating sterile intersexes.

Determining sex is one of the most fundamental developmental decisions that most organisms make. In many species, sex is determined by a mechanism that utilizes specialized sex chromosomes. In humans, the Y chromosome induces male development in XY embryos, and its absence in XX embryos elicits female development (Figure 1A) (Brush 1978). In other species, sex is specified by a mechanism that distinguishes one X chromosome from two: 2X embryos become females, while 1X embryos become males (Figure 1A). The nematode *Caenorhabditis elegans*, like the fruit fly *Drosophila melanogaster*, determines sex with high fidelity by tallying X-chromosome number relative to ploidy, the sets of autosomes (X:A signal) (Bridges 1921; Nigon 1951). In nematodes, the process is executed with remarkable precision: embryos with ratios of 1X:2A (0.5) or 2X:3A (0.67) develop into fertile males, while embryos with ratios or 3X:4A (0.75) or 2X:2A (1.0) develop into self-fertile hermaphrodites (Figure 1B) (Nigon 1951).

Chromosome-based mechanisms of sex determination have the potential to cause an imbalance in X-linked gene products between the sexes. As a consequence, many organisms reliant on such chromosomal mechanisms have co-evolved a dosage compensation process to balance X-chromosome gene expression between the sexes (Figure 1A). Strategies for dosage compensation differ from worms to mammals, but invariably a regulatory complex is targeted to X chromosomes of one sex to modulate transcription along the entire chromosome. Human females inactivate one of their two X chromosomes (Jegu *et al.* 2017; Galupa and Heard 2018), fruit fly males of *D. melanogaster* double the transcription from their single X chromosome (Lucchesi and Kuroda 2015; Jordan *et al.* 2019), and nematode hermaphrodites of *C. elegans* reduce transcription from both X chromosomes by approximately half (Meyer 2018) so that X-chromosome gene expression is balanced between the two sexes (Figure 1A). Failure to achieve dosage compensation causes sex-specific lethality. Human females die without X inactivation, male fruit flies die without elevated X transcription, and hermaphrodite nematodes die without reduced X transcription. In nematodes, as in flies, sex determination and dosage compensation are linked through a master sex-determination switch gene that coordinately controls both processes and is regulated directly by the X:A signal. Hence, failure to count X chromosomes accurately to decide between alternative sexual fates causes death.

At the outset of studies to determine mechanisms that underlie X-chromosome counting, the nematode community had no knowledge of whether *C. elegans* utilized a dosage compensation process to compensate for the difference in X-chromosome dose, and if it did, whether this process was linked to the sex-determination decision itself. If it were, the phenotype caused by disrupting the X:A signal might be sex-specific lethality due to improper X gene expression, thus masking reversal of sexual fate. Therefore, early work to discover the basis of the primary sex-determining signal began by establishing that an X-chromosome dosage compensation process functions in *C. elegans* to balance expression and also by determining that the failure to compensate causes hermaphrodite-specific lethality due to overexpression of X-linked genes. These results revealed that incorrect X-chromosome counting might also cause sex-specific lethality in the worm. Subsequent studies showed that sex determination and dosage compensation are indeed coordinately regulated by a set of hermaphrodite-specific genes that activate the dosage compensation mechanism in XX embryos and also repress the male program of sexual differentiation. These genes, in turn, are controlled by a male-specific master sex-determination gene that is the immediate target of the X:A signal. This review first addresses mechanisms underlying X-chromosome counting and then X-chromosome dosage compensation.

## The X-chromosome counting mechanism that determines sex

### *xol-1* is the direct gene target of the X:A signal

**Figure 2:**
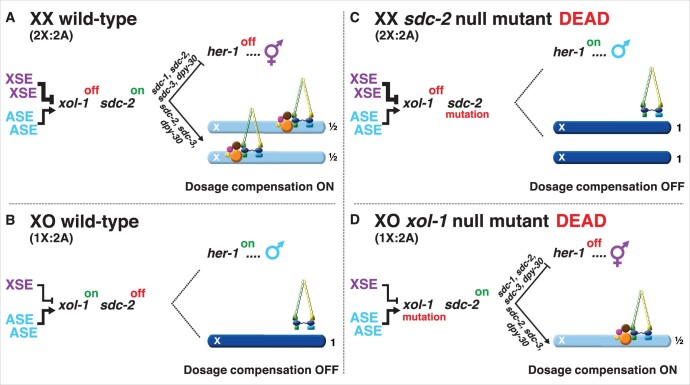
Overview of the X:A signal and the regulatory hierarchy that controls nematode sex determination and dosage compensation. (A, B) In wild-type animals, the X:A signal that determines sexual fate is a competition between a set of genes on X called XSEs that represses their direct gene target *xol-1* (XO lethal) in a cumulative dose-dependent manner via transcriptional and post-transcriptional mechanisms and a set of genes on autosomes called ASEs that stimulate *xol-1* transcription in a cumulative dose-dependent manner. *xol-1* is the master sex-determination switch gene that must be activated in XO animals to set the male fate and must be repressed in XX animals to permit the hermaphrodite fate. (A) Two doses of XSEs in diploid XX animals win out and repress *xol-1*, but (B) the single dose of XSEs in diploid XO animals does not turn *xol-1* off. (B) *xol-1* triggers male sexual development in wild-type XO animals by repressing the feminizing switch gene *sdc-2* (sex determination and dosage compensation). (A) Together with *sdc-1, sdc-3*, and *dpy-30*, the *sdc-2* gene induces hermaphrodite sexual development in XX animals by repressing the male sex-determining gene *her-1.* Together with *sdc-3* and *dpy-30*, *sdc-2* triggers binding of a dosage compensation complex (DCC) onto both hermaphrodite X chromosomes to repress gene expression by half. *sdc-1* is essential for DCC activity, but not for loading of the DCC onto X. The DCC is a condensin complex that restructures the topology of X. (C) *sdc-2* mutations kill XX animals by prevent the DCC from binding to X chromosomes, resulting in overexpression of X-linked genes. The mutations also masculinize XX animals, because *her-1* is not repressed. (D) Loss-of-function *xol-1* mutations enable *sdc-2* to be active and permit the DCC to bind the single male X, thereby killing XO animals from reduced X-chromosome expression. The dying *xol-1* XO mutant animals are feminized because *her-1* is repressed. Hence, mutations that disrupt elements of the X:A signal itself transform sexual fate, but also kill due to altered X-chromosome gene expression.

**Figure 3:**
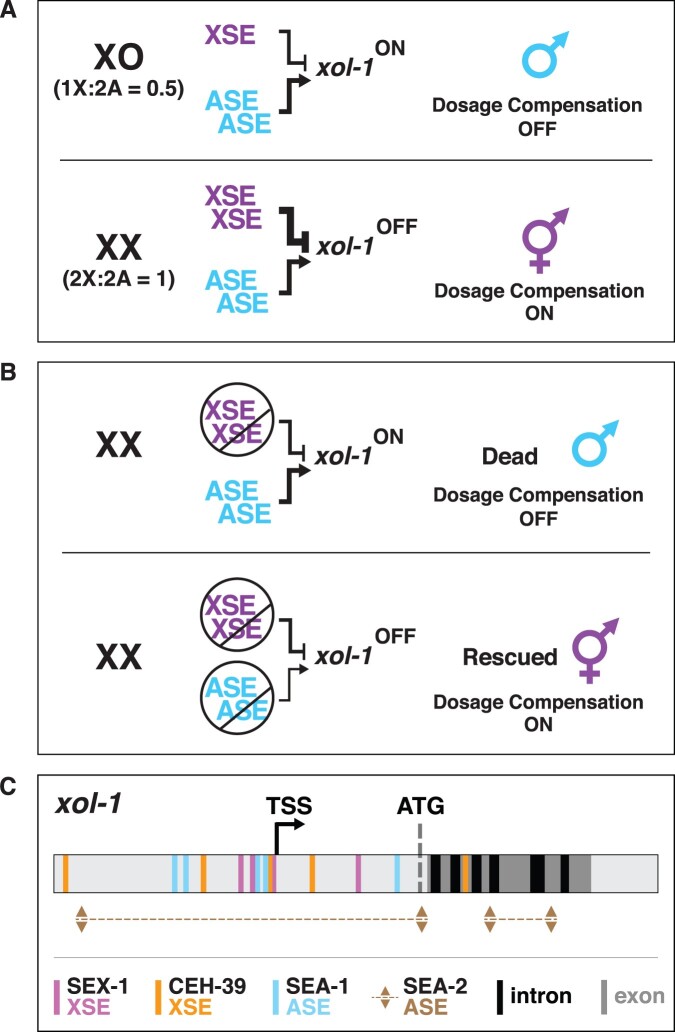
Dissecting the X:A sex determination signal. (A) XSE regulate *xol-1* in a dose-dependent manner in the context of two doses of ASE. Two doses of XSEs win out and repress *xol-1* in diploid animals with two doses of ASE, which stimulate *xol-1* expression. One XSE dose does not prevail in repressing. When *xol-1* is activated in 1X:2A animals, the dosage compensation machinery is turned off. XO animals are viable and develop as males. When *xol-1* is repressed in 2X:2A animals, the dosage compensation machinery is activated, thereby reducing X-linked gene expression by half. XX animals are viable and develop as hermaphrodites. (B) Loss-of-function mutations in XSEs were identified in genetic screens because they caused a *xol-1* reporter transgene to be activated in XX animals, resulting in the masculinization and death of XX animals. XSEs were also discovered as suppressors of the male lethality caused by duplication of large regions of X. Loss-of-function mutations in ASEs were identified in genetic screens because they suppressed the lethality of mutations in XSEs and prevented the transformation of sexual fate caused by them. (C) Locations of binding sites in the 5′ *xol-1* regulator regions for the XSEs (SEX-1 and CEH-39) that repress *xol-1* transcription and the ASEs (SEA-1 and SEA-2) that activate *xol-1* transcription. The general regions of SEA-2 binding were defined but not yet the precise binding sites. SEX-1 is a nuclear hormone receptor; CEH-39 is a ONECUT homeodomain protein; SEA-1 is a T-box protein; SEA-2 is a zinc-finger protein. After the molecular tug-of-war to control *xol-1* transcription, a second tier of regulation occurs to control *xol-1* pre-mRNA splicing by the XSE FOX-1 (see Figure  5).

The X:A signal determines sex in *C. elegans* by regulating *xol-1* (XO lethal), the master sex-determination switch gene that sets the male fate (Figures 2, A and B and 3A; Table 1) (Miller *et al.* 1988; Akerib and Meyer 1994; Rhind *et al.* 1995; Carmi *et al.* 1998). *xol-1* controls not only the choice of sexual fate but also the level of X-chromosome gene expression by controlling the process of X-chromosome dosage compensation (Miller *et al.* 1988; Rhind *et al.* 1995). Understanding *xol-1* function was pivotal to dissecting the X:A signal. *xol-1* encodes a GHMP kinase that must be activated to trigger the male fate and repressed to permit the hermaphrodite fate (Luz *et al.* 2003). *xol-1* directs male development in XO embryos by repressing the XX-specific gene *sdc-2* (sex determination and dosage compensation), which encodes a 350 kDa protein with no known homology (Figure 2B) (Miller *et al.* 1988; Nusbaum and Meyer 1989; Rhind *et al.* 1995; Dawes *et al.* 1999). *sdc-2* directs hermaphrodite sexual differentiation in XX embryos by repressing transcription of the male sex-determining gene *her-1* (*her*maphrodization) (Nusbaum and Meyer 1989; Chu *et al.* 2002). *sdc-2* also activates dosage compensation in XX embryos by triggering binding of a dosage compensation complex (DCC) to both X chromosomes, where it reduces transcription by approximately half and thereby balances X expression with that from the single X of XO males (1X:2A) (Figure 2A) (Dawes *et al.* 1999).

**Table 1:** Genes central to sex determination and dosage compensation

Gene	Molecular identity	Gene function	Mutant phenotype
*sex-1*	Nuclear hormone receptor	X signal element Represses *xol-1* transcription	XX-specific lethal and masculinized

*ceh-39*	ONECUT homeodomain protein	X signal element Represses *xol-1* transcription	XX-specific lethal and masculinized with other XSE mutations

*fox-1*	RNA binding protein with an RRM domain that recognizes GCAUG and GCACG motifs	X signal element Causes nonproductive *xol-1* pre-mRNA splicing	XX-specific lethal and masculinized with other XSE mutations

*sea-1*	T-box transcription factor	Autosomal signal element Activates *xol-1* transcription	Suppresses XX-specific lethality of *ceh-39 sex-1* XX mutants

*sea-2*	Zinc-finger protein	Autosomal signal element Activates *xol-1* transcription	Suppresses XX-specific lethality of *ceh-39 sex-1* XX mutants

*xol-1*	GHMP kinase	Direct target of X:A signal Master sex determination switch gene in XO animals that specifies male fate and turns off the dosage compensation process by repressing *sdc-2*	XO-specific lethal and feminized

*sdc-1*	Zinc-finger protein DCC subunit	Controls sex determination and dosage compensation in XX animals by acting with *sdc-2* DCC activity	Dpy and masculinized XX animals

*sdc-2*	No homology found 350 kDa protein DCC subunit	Master sex determination switch gene in XX animals that specifies hermaphrodite fate by repressing *her-1* and by triggering binding of the dosage compensation complex to X chromosomes DCC activity	XX-specific lethal and masculinized

*sdc-3*	Zinc-finger protein with ATP binding domain DCC subunit	Controls sex determination and dosage compensation in XX animals by acting with *sdc-2* DCC activity	XX-specific lethal Some classes of alleles cause masculinization

*dpy-30*	Subunit of human and *C. elegans* histone H3K4 methyltransferase complexes *C. elegans* DCC subunit	Controls sex determination and dosage compensation in XX animals Controls general gene expression in XX and XO animals. COMPASS complex activity DCC activity	100% XX lethal 20% XO lethal Viable XO animals are scrawny, developmentally delayed, and mating defective due to aberrant tail morphology
*dpy-21*	Jumonji H4K20me2 demethylase DCC subunit	Controls dosage compensation in XX animals Converts H4K20me2 to H4K20me1 on somatic X chromosomes and meiotic autosomes DCC activity	XX-specific Dpy Expanded meiotic autosome axis length

*dpy-26*	Homolog of human condensin subunit CAP-H *C. elegans* condensin I subunit DCC condensin subunit Kleisin	Controls dosage compensation in XX animals Controls structure and segregation of mitotic and meiotic chromosomes in XX and XO animals Condensin I activity DCC activity	XX-specific lethal High incidence of males Moderate defect in mitotic and meiotic chromosome segregation in XX and XO animals Altered meiotic crossover recombination and expanded meiotic chromosome axis length

*dpy-27*	Homolog of human condensin subunit SMC4 DCC condensin subunit ATPase	Controls dosage compensation in XX animals DCC activity	XX-specific lethal

*dpy-28*	Homolog of human condensin subunit CAP-D2 *C. elegans* condensin I subunit DCC condensin subunit HEAT repeat	Controls dosage compensation in XX animals Controls structure and segregation of mitotic and meiotic chromosomes in XX and XO animals Condensin I activity DCC activity	XX-specific lethal High incidence of males Moderate defect in mitotic and meiotic chromosome segregation in XX and XO animals Altered meiotic crossover recombination and expanded meiotic chromosome axis length.

*capg-1*	Homolog of human condensin subunit CAP-G *C. elegans* condensin I subunit DCC condensin subunit HEAT repeat	Controls dosage compensation in XX animals Controls structure and segregation of mitotic and meiotic chromosomes in XX and XO animals Condensin I activity DCC activity	XX-specific lethal Moderate defect in mitotic and meiotic chromosome segregation in XX and XO animals Altered meiotic crossover recombination and expanded meiotic chromosome axis length

*mix-1*	Homolog of human condensin subunit SMC2 Subunit of *C. elegans* condensin I and condensin II complexes DCC condensin subunit ATPase	Controls dosage compensation in XX animals Controls structure and segregation of mitotic and meiotic chromosomes in XX and XO animals Condensin I and condensin II activity DCC activity	Lethal to both XX and XO animals Severe defect in mitotic and meiotic chromosome segregation Defective in dosage compensation Altered meiotic crossover recombination and expanded meiotic chromosome axis length

If *xol-1* function is disrupted in diploid XO animals either by mutation or by inappropriate repression, *sdc-2* becomes activated, the DCC binds to the single X chromosome and kills all males by reducing X expression (Figure 2D) (Miller *et al.* 1988; Akerib and Meyer 1994; Rhind *et al.* 1995; Chuang *et al.* 1996; Carmi and Meyer 1999). *her-1* is repressed, and the dying XO animals become feminized (Figure 2D). Conversely, if *sdc-2* is mutated in diploid XX animals or inappropriately repressed because *xol-1* becomes activated, the DCC does not bind to X chromosomes, and all hermaphrodites die from elevated X expression (Figure 2C) (Nusbaum and Meyer 1989; Chuang *et al.* 1996; Dawes *et al.* 1999). The dying XX animals are also masculinized.

Mutations that increase the repressive X:A signal kill XO animals but have no effect on XX animals, like mutations in *xol-1* (Akerib and Meyer 1994; Rhind *et al.* 1995; Carmi *et al.* 1998; Carmi and Meyer 1999). In contrast, mutations that decrease the repressive X:A signal kill XX animals, but have no effect on XO animals, like mutations in *sdc-2* (Figure 2C) (Akerib and Meyer 1994; Carmi *et al.* 1998; Carmi and Meyer 1999; Dawes *et al.* 1999). Thus, incorrect assessment of the sex signal causes not only transformation of sexual fate but also sex-specific lethality.

### Dose-sensitive signals relay doses of X chromosomes and autosomes to determine sex

Genetic and molecular experiments revealed that a set of genes on X chromosomes called X-signal elements (XSEs) communicates X-chromosome dose by repressing *xol-1* in a cumulative, dose-dependent manner (Figure 3A;Table 1) (Akerib and Meyer 1994; Hodgkin *et al.* 1994; Nicoll *et al.* 1997; Carmi *et al.* 1998; Carmi and Meyer 1999; Skipper *et al.* 1999; Gladden and Meyer 2007; Farboud *et al.* 2013, 2020). The distinguishing genetic feature of XSEs is the reciprocal, sex-specific phenotypes caused by changing their dose in XX *vs* XO diploid animals. Decreasing XSE dose kills XX hermaphrodites, but not XO males, by inappropriately activating *xol-1* in XX embryos (Figure 3B). Increasing XSE dose kills XO males, but not XX hermaphrodites, by inappropriately repressing *xol-1* in XO embryos.

Initial evidence for XSEs came from the identification of relatively large X-chromosome duplications derived from the left end of X that caused XO-specific lethality (Akerib and Meyer 1994; Hodgkin *et al.* 1994; Hodgkin and Albertson 1995). The lethality was suppressed by mutations in *sdc-2*. These findings suggested that the duplications supplied extra copies of X loci that comprised at least part of the X component of the X:A signal and that duplication of these X loci was sufficient to repress *xol-1* in XO animals and hence kill them. The first specific XSE, named *fox-1* (feminizing locus on X), was discovered through mutations that suppressed the XO lethality caused by the large X duplications (Akerib and Meyer 1994; Hodgkin *et al.* 1994). Additional XSEs were identified through suppression of the XO lethality caused by X duplications (Gladden and Meyer 2007) or by directed genetic screens for mutations that activated expression of a *lacZ* reporter transgene driven by the *xol-1* promoter in XX embryos (Carmi *et al.* 1998).

**Figure 4:**
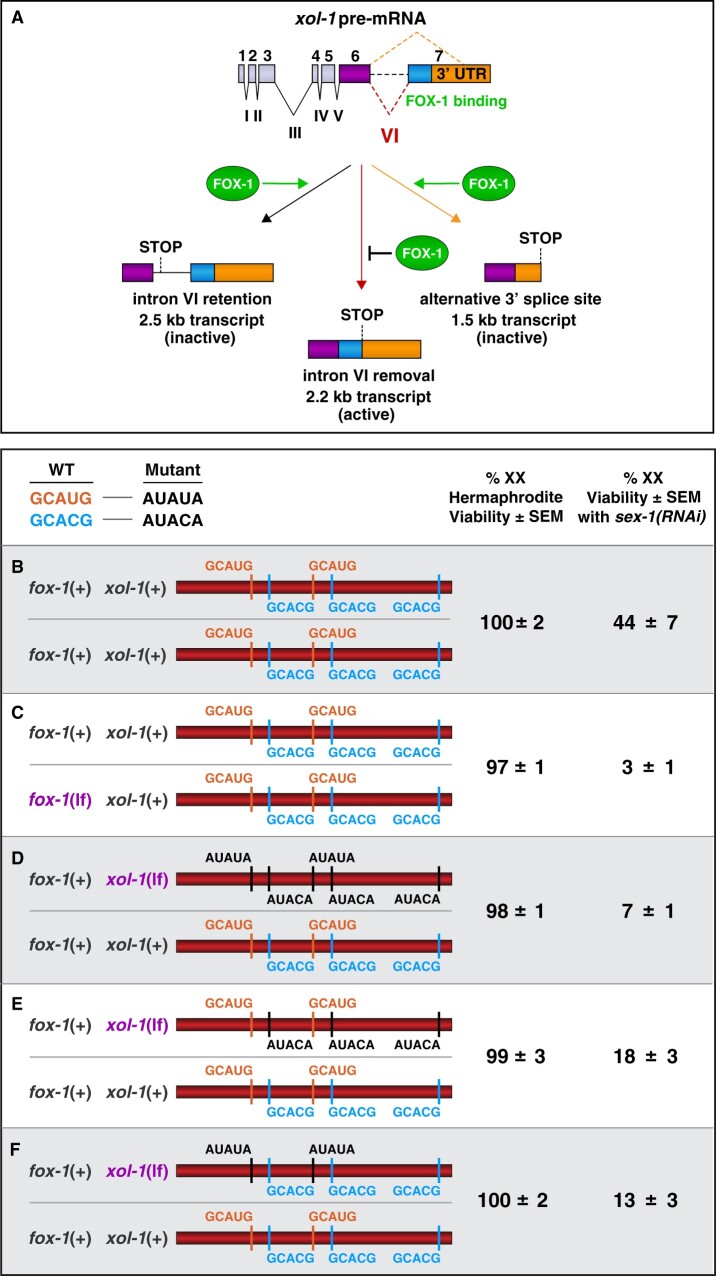
Dose-dependent pre-mRNA splicing regulation of *xol-1* by the RNA binding protein FOX-1, an XSE. (A) Summary of *xol-1* splicing regulation by FOX-1. By binding to multiple GCAUG and GCACG motifs in intron VI of *xol-1*, FOX-1 reduces formation of the male-determining 2.2 kb transcript by causing intron VI retention (2.5 kb transcript) or by directing use of an alternative 3′ splice acceptor site, causing deletion of essential exon 7 coding sequences (blue) and part of the 3′ UTR (orange) (1.5 kb transcript). (B–F) Two copies of *fox-1*(+) and multiple high-affinity GCAUG and GCACG motifs in both copies of intron VI are essential for FOX-1-mediated alternative splicing of *xol-1* in XX animals. DNA sequences in the upper left compare the wild-type *vs* mutant versions of FOX-1 binding motifs in *xol-1* intron VI that were used to assess regulation by FOX-1. Diagrams on the left show sequences of FOX-1 binding motifs in either a *fox-1* mutant or in animals carrying heterozygous combinations of mutant motifs in *xol-1* intron VI. Viability of XX progeny were assayed in each cohort of *sex-1*(+) or *sex-1(RNAi)* XX animals to assess the dose-dependence of FOX-1 action in regulating *xol-1* splicing. Percentages on the right reflect the viability of *sex-1*(+) or *sex-1(RNAi)* XX animals with different heterozygous combinations of intron VI or *fox-1* mutations. None of the heterozygous mutant combinations in intron VI or *fox-1* affects the viability of *sex-1*(+) animals, but they have strong effects on *sex-1(RNAi)* animals. (B, C) Mutating one copy of *fox-1* reduced viability of *sex-1(RNAi)* XX animals from 44% to 3%. (D) Similarly, mutating one copy of all GCAUG and GCACG motifs in one intron reduced viability to 7%. Mutating only one copy of the three GCACG motifs (E) or one copy of the two GCAUG motifs (F) in one intron had an intermediate effect, resulting in 18% or 13% viability. Thus, FOX-1 acts in a dose-dependent manner to regulate *xol-1* splicing in XX *vs* XO animals.

Extensive genetic, molecular, and biochemical analysis revealed that XSEs repress *xol-1* via two distinct mechanisms: transcriptional regulation through the nuclear hormone receptor SEX-1 (Signal Element on X) and the ONECUT homeodomain protein CEH-39 (*C. elegans*Homeobox) (Figure 3C) (Carmi *et al.* 1998; Carmi and Meyer 1999; Gladden *et al.* 2007; Gladden and Meyer 2007; Farboud *et al.* 2013) and pre-mRNA splicing regulation through the RNA binding protein FOX-1 (Figure 4A) (Nicoll *et al.* 1997; Skipper *et al.* 1999; Farboud *et al.* 2020). Genetically, these three XSEs act synergistically: mutating only one of the two copies of all three XSEs in XX animals causes extensive hermaphrodite-specific lethality. The XX lethality is suppressed by *xol-1* mutations. Reciprocally, adding only one extra copy of all three XSEs to XO animals causes extensive male-specific lethality. The XO lethality is suppressed by *sdc-2* mutations. Thus, having two doses of XSEs in XX embryos is as important for viability as restricting the dose of XSEs to one in XO embryos.

The dose effect of XSEs occurs in the context of a set of genes on autosomes called autosomal-signal elements (ASEs) that communicates ploidy by stimulating *xol-1* activity in a dose-dependent manner to counter XSEs (Figure 3A;Table 1) (Powell *et al.* 2005; Farboud *et al.* 2013). ASEs were discovered through loss-of-function mutations that suppressed the XX-specific lethality caused by loss-of-function mutations in XSEs (Figure 3B) (Powell *et al.* 2005; Farboud *et al.* 2013). The distinguishing genetic feature of ASEs is that decreasing ASE dose is detrimental to XO males but not XX hermaphrodites because it fails to stimulate *xol-1* activity, while increasing ASE dose is detrimental to XX hermaphrodites but not XO males because it activates *xol-1* (Powell *et al.* 2005; Farboud *et al.* 2013).

### Transcriptional repression by XSEs

Direct DNA-binding studies performed *in vitro* revealed that the two known XSE transcriptional repressors, the nuclear hormone receptor SEX-1 and the ONECUT homeodomain protein CEH-39 bind directly to multiple, distinct nonoverlapping sites in the 5′ regulatory regions of *xol-1* in XX embryos to repress *xol-1* transcription (Figure 3C) (Farboud *et al.* 2013). In contrast, the two ASE transcriptional activators, the T-box transcription factor SEA-1 (Signal Element on Autosome), and the zinc-finger protein SEA-2 bind directly to multiple, nonoverlapping sites in *xol-1* to activate transcription (Figure 3C) (Farboud *et al.* 2013).

In complementary experiments performed *in vivo*, mutating combinations of XSE and ASE binding sites on *xol-1* transgenes in XX strains carrying endogenous *xol-1* deletions recapitulated the misregulation of *xol-1* transcription caused by disrupting the corresponding ASE or ASE genes. For example, mutating all SEX-1 and CEH-39 binding sites in both copies of the *xol-1* transgene killed all XX animals, and double mutations in *sea-1* and *sea-2* suppressed the XX lethality (Farboud *et al.* 2013). Furthermore, deletion of all SEA-1 binding sites in the *xol-1* transgene suppressed the XX lethality of *sex-1(null)* mutants. Thus, XSEs and ASEs antagonize each other’s opposing transcriptional activities to control *xol-1* transcript levels (Farboud *et al.* 2013). The X:A signal is transmitted in part through multiple antagonistic molecular interactions carried on a single promoter to regulate transcription.

In addition to its primary role of transmitting X-chromosome dose to determine sex, SEX-1 plays roles in DNA-damage response and radiation protection (van Haaften *et al.* 2006). *sex-1(RNAi)* increased sensitivity to ionizing radiation and to the drugs methyl methanesulfonate and camptothecin. *sex-1(RNAi)* caused cells of the mitotic germline to fail to arrest following ionizing radiation and blocked apoptosis in the meiotic germline (van Haaften *et al.* 2006). In addition, SEX-1 functions with other nuclear hormone receptors in a metabolic gene regulatory network (Arda *et al.* 2010).

### Splicing regulation by XSEs

**Figure 5:**
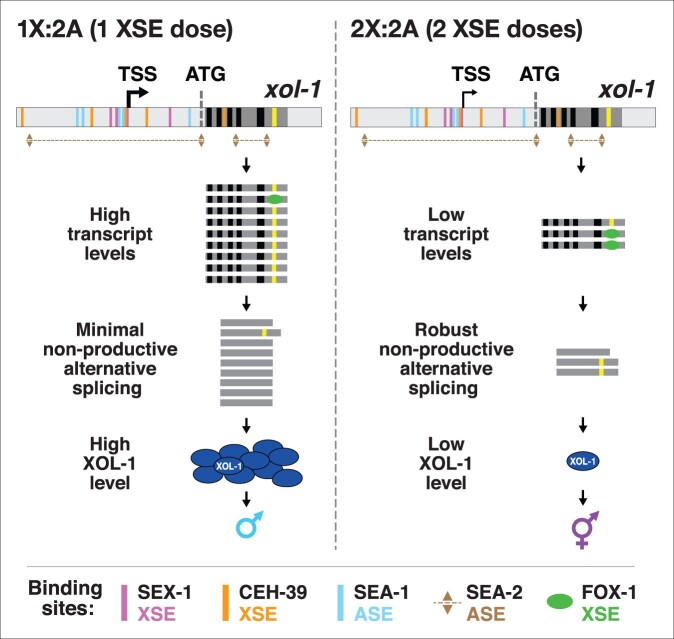
Model for X:A signal assessment: two tiers of *xol-1* repression. XSEs and ASEs bind directly to numerous nonoverlapping sites in the 5′ regulatory region of *xol-1* to antagonize each other’s opposing transcriptional activities and thereby control *xol-1* transcription. Molecular rivalry at the *xol-1* promoter between the XSE transcriptional repressors and ASE transcriptional activators causes high *xol-1* transcript levels in 1X:2A embryos with one dose of XSEs and low levels in 2X:2A embryos with two doses of XSE. In a second tier of *xol-1* repression, the XSE RNA binding protein FOX-1 then enhances the fidelity of X-chromosome counting by binding to numerous GCAUG and GCAUG motifs in intron VI (yellow) of the residual *xol-1* pre-mRNA, thereby causing nonproductive alternative splicing and hence *xol-1* mRNA variants that have in-frame stop codons or lack essential exons. High XOL-1 protein induces the male fate and low XOL-1 permits the hermaphrodite fate. Light gray rectangles represent 5′ and 3′ *xol-1* regulatory regions, dark gray rectangles represent *xol-1* exons, black rectangles represent unregulated *xol-1* introns, and the yellow rectangle represents the alternatively spliced intron VI regulated by FOX-1. The orange rectangle that represents a CEH-39 binding site in the gene body is located in an exon.

Fidelity of X:A signaling is enhanced by a second tier of dose-dependent *xol-1* repression via the RNA binding protein FOX-1, which has an RNA Recognition Motif (RRM) (Figures 4A and 5) (Nicoll *et al.* 1997; Carmi and Meyer 1999; Skipper *et al.* 1999; Farboud *et al.* 2020). It acts on residual *xol-1* transcripts present in diploid XX animals after *xol-1* repression by XSE transcription factors. FOX-1 is the founding member of an ancient family of sequence-specific RNA binding proteins conserved from worms to humans, and its first defined function was in *C. elegans* sex determination (Akerib and Meyer 1994; Hodgkin *et al.* 1994; Nicoll *et al.* 1997; Skipper *et al.* 1999; Conboy 2017). FOX proteins regulate diverse aspects of RNA metabolism in different species, including alternative pre-mRNA splicing, mRNA stability, translation, micro-RNA processing, and transcription (Jin *et al.* 2003; Ray *et al.* 2013; Kim *et al.* 2014; Carreira-Rosario *et al.* 2016; Chen *et al.* 2016; Lee *et al.* 2016; Wei *et al.* 2016; Conboy 2017; Farboud *et al.* 2020). FOX proteins act as developmental regulators to control neuronal and brain development and muscle formation in vertebrates (Shibata *et al.* 2000; Underwood *et al.* 2005; Gehman *et al.* 2011, 2012; Singh *et al.* 2014; Gao *et al.* 2016; Lee *et al.* 2016; Wei *et al.* 2016; Begg *et al.* 2020) and in *C. elegans* (Kuroyanagi *et al.* 2006, 2007, 2013).

For its role in *C. elegans* sex determination, FOX-1 triggers hermaphrodite development in XX nematode embryos by regulating alternative *xol-1* pre-mRNA splicing to inhibit formation of the mature 2.2 kb transcript that is both necessary and sufficient for male-determining *xol-1* activity in XO animals (Figure 4A) (Nicoll *et al.* 1997; Skipper *et al.* 1999; Farboud *et al.* 2020). Experiments performed *in vivo* demonstrated that intron VI alone mediates FOX-1-directed splicing repression of endogenous *xol-1* and can confer FOX-1-directed alternative splicing regulation onto integrated *lacZ* reporters (Farboud *et al.* 2020). Deleting intron VI from the endogenous *xol-1* gene prevented FOX-1 from blocking proper *xol-1* splicing, resulting in elevated 2.2 kb transcript levels in XX embryos. Inserting intron VI into *lacZ* enabled FOX-1 to block removal of the ectopic intron, thereby preventing *lacZ* expression.

RNA-binding studies *in vitro* together with genome editing experiments *in vivo* demonstrated that combining multiple RNA binding motifs in the *xol-1* gene target with a twofold change in FOX-1 concentration between XX and XO embryos achieves dose-sensitivity in spicing regulation to determine sex (Farboud *et al.* 2020). FOX-1 binds to two GCAUG and three GCACG motifs in intron VI. Both motifs are utilized in mammalian cell lines for mammalian Rbfox-mediated splicing regulation, although only one copy of a motif is needed, because Rbfox has a tyrosine-rich, low-complexity domain that nucleates its own aggregation to reach an appropriate concentration of bound proteins (Ying *et al.* 2017). *Caenorhabditis* *elegans* FOX-1 binding to the five GCAUG and GCACG motifs in *xol-1* pre-mRNA causes either intron retention to produce a 2.5 kb transcript with an in-frame stop codon, or promotes use of an alternative 3′ splice site to produce a 1.5 kb transcript that deletes essential exon coding sequences (Figure 4A). Either alternative splicing event precludes formation of male-determining XOL-1 protein in XX animals.

Experiments using a sensitized mutant background in which *xol-1* transcripts were partially elevated in XX animals revealed the dose-sensitive action of FOX-1 binding motifs (Figure 4B). Mutating different combinations of endogenous GCAUG and GCACG motifs in intron VI using genome editing to create AUAUA and AUACA motifs, respectively, reduced nonproductive splicing and enhanced XX-specific lethality caused by *sex-1(RNAi)*, but did not cause lethality in otherwise wild-type XX animals (Figure 4, B–F) (Farboud *et al.* 2020). Splicing regulation is dose-dependent: mutating one copy of *fox-1* or all five binding motifs in one copy of *xol-1* kills almost all XX animals sensitized by reduced XSE activity from *sex-1(RNAi)* (Figure 4, C and D). Mutating one copy of the three GCACG motifs or one copy of the two GCAUG motifs in *sex-1(RNAi)* XX animals caused an intermediate level of XX-specific lethality (Figure 4, E and F). Thus, use of multiple high-affinity RNA binding sites in a *xol-1* intron permits the level of FOX-1 protein produced from two copies of *fox-1* in XX embryos to reach the threshold necessary to block formation of properly spliced male-determining *xol-1* transcripts and hence inhibit XOL-1 production (Farboud *et al.* 2020).

The combination of transcriptional regulation and pre-mRNA splicing regulation is critical to convert the twofold difference in levels of *xol-1* regulators between XO an XX embryos into a robust on/off binary switch that specifies sexual fate with high fidelity (Figure 5). Variability and imprecision in transcriptional repression caused by the small difference in concentration of X-linked transcriptional repressors between XO and XX embryos can be compensated by the subsequent dose-sensitive splicing regulation that blocks formation of the active *xol-1* splice variant in XX embryos. Neither mechanism alone is sufficient to repress *xol-1* reliably in XX embryos with only the twofold difference in XSE regulatory factors between the sexes.

Strikingly, the process of dosage compensation affects expression of the very XSEs that control it. XSEs become dosage compensated once sex is determined (Gladden *et al.* 2007). The X:A signal becomes roughly equivalent in XO and XX animals. Hence sexual differentiation and dosage compensation must become controlled by genes downstream of *xol-1* in the sex-determination and dosage compensation pathways.

Dissecting the molecular mechanisms underlying X-chromosome counting has revealed how small quantitative differences in intracellular signals can be translated into dramatically different fates. Molecular knowledge of the XSE repressors and ASE activators of *xol-1* and their binding targets lays the foundation for discovering the precise nature of their molecular competition to regulate this master sex-determination switch gene. Future research will also determine when and where during embryogenesis the X:A signal must be assessed to turn *xol-1* on in XO animals and off in XX animals. Must all cells assess the X:A signal independently and specify the same choice to establish proper sexual fate, or is the fidelity of the sex-determination decision critical in only a subset of cells that then dictates the sexual fate of other cells? What kinetic and threshold levels of *xol-1* expression in XO embryos and *sdc-2* expression in XX embryos are required to enable development into fully fertile males or hermaphrodites?

### Comparison of X:A counting mechanisms between worms and flies

Like *C. elegans*, the fruit fly *D. melanogaster* utilizes the combination of transcriptional and pre-mRNA splicing regulation to enhance the precision of X:A counting when controlling the direct target of the X:A signal: *Sxl* (*Sex-lethal*), a master sex-determination switch gene. *Sxl* encodes an X-linked RNA binding protein with an RRM domain that dictates female development when active and permits male development when inactive (Cline and Meyer 1996). SXL protein elicits female sexual differentiation by directing proper pre-mRNA splicing of its downstream sex-determination target gene called *tra* (transformer), a switch gene essential for female development (Sosnowski *et al.* 1989; Inoue *et al.* 1990; Valcarcel *et al.* 1993). SXL also sets the level of X-chromosome gene expression by regulating fly dosage compensation (Cline and Meyer 1996). In females, SXL prevents assembly of the male-specific DCC (MSL complex) by blocking proper splicing of an essential MSL subunit (Lucchesi and Kuroda 2015). The MSL complex binds the single male X to increase its transcription.

*Sxl* is activated in 2X:2A embryos, but not 1X:2A embryos, by a set of feminizing XSEs that stimulate transcription of *Sxl* in a dose-dependent manner. Only the double dose of X-linked transcription factor XSEs in 2X:2A embryos along with the double dose of *Sxl* itself can reliably turn *Sxl* on. Once produced, SXL protein functions in a positive autoregulatory loop to control splicing of its own pre-mRNA in a dose-dependent manner and thereby promotes continued production of female-specific SXL protein independently of the initiating signal (Bell *et al.* 1991; Sakamoto *et al.* 1992; Horabin and Schedl 1993; Cline and Meyer 1996). By binding to two neighboring *Sxl* introns, SXL protein prevents inclusion of the intervening male-specific exon 3. This male exon encodes an in-frame stop codon that prevents translation of the full-length female SXL protein when incorporated into mature RNA. Thus, although *Sxl* responds to fly XSEs to determine sex, its location on X and its auto-regulatory feature allow it to serve as both signal and target.

In contrast to worms, ploidy does not appear to be signaled in flies by a corresponding set of masculinizing ASE genes. Instead, the major effect of ploidy in this dose-sensitive process is indirect, influencing the timing of cellularization during early development and thereby the length of time during which XSE protein can increase in concentration to reach the threshold necessary to trigger the *Sxl* autoregulatory feedback loop (Erickson and Quintero 2007; Salz and Erickson 2010)*.* The lower the ploidy, the later the embryos cellularize, the longer the XSEs and *Sxl*’s own product can accumulate, and the higher the probability of stably activating *Sxl*. As a consequence, 1X:1A embryos become females instead of males, and 2X:3A embryos become mosaic intersexes, unlike nematodes where 2X:3A embryos can become fertile males. Only a single fly ASE was identified through extensive genetic screens to identify suppressors of XSE mutations (Cline and Meyer 1996). That ASE acts as a weak transcriptional repressor of *Sxl* that fine-tunes the counting process in diploids.

## X-chromosome dosage compensation

### Discovery of a dosage compensation process

Gene expression in metazoans is controlled by diverse regulatory mechanisms that act over dramatically different distances (Ghosh and Meyer 2021). While regulatory mechanisms that act locally on individual genes are reasonably well understood, a major challenge persists in understanding the mechanisms that coordinately regulate gene expression over large chromosomal territories and the functional relationship between chromatin modification and chromosome structure in this long-range gene regulation. The study of X-chromosome gene regulation during dosage compensation is advantageous for understanding these connections. Dosage compensation regulates thousands of genes simultaneously, it distinguishes X chromosomes from autosomes, it discriminates between the sexes in modulating gene expression along X, and it utilizes histone modifications as well as chromosome structure to modulate gene expression across X.

Initial evidence that *C. elegans* might employ a dosage compensation mechanism came from the discovery of genes whose mutant phenotypes depended on X-chromosome dose. Sex-specific mutations were found that preferentially killed or enfeebled XX animals but caused no phenotype in XO animals (Hodgkin 1983; Meyer and Casson 1986). The fact that sex-specific lethality occurred whether the XX animals were hermaphrodites or sexually transformed into males by mutation of a hermaphrodite-determining gene showed that it was X-chromosome dose that mattered, not the sex per se. Rare XX animals that escaped lethality were dumpy (Dpy), and most were infertile.

For the putative dosage compensation genes *dpy-26*, *dpy-27*, and *dpy-28*, virtually all XX homozygous mutant progeny from homozygous null mutant mothers (m^−^z^−^) were dead; XO animals were viable (Table 1) (Hodgkin 1983; Meyer and Casson 1986; Plenefisch *et al.* 1989). The maternal product from the heterozygous mothers was sufficient to permit the first generation of homozygous mutant XX animals (m^+^z^−^) to be viable and fertile. The maternal contribution enables dosage compensation to be enacted rapidly after assessment of the X:A signal. It also enables the genes to be utilized for germline functions such as chromosome segregation.

In contrast, loss-of-function mutations in the gene *dpy-21* caused a less severe XX-specific phenotype: a recessive Dpy phenotype with little lethality and no maternal effect in XX mutants (Table 1) (Hodgkin 1983; Meyer and Casson 1986; Plenefisch *et al.* 1989). XO mutants appeared wildtype. The sensitivity of *dpy-21* mutants to X dosage was also apparent from the response to adding extra doses of X (Hodgkin 1983). *dpy-21* mutants were killed by one extra X (3X : 2A *dpy-21* mutants), whereas two extra X chromosomes were needed to kill an otherwise wild-type diploid worm [4X:2A *dpy-21*(+)]. Only one extra X caused the 3X:2A *dpy-21*(+) animals to be Dpy. These genetic observations suggested that the *dpy* mutations might cause an elevation in X-chromosome gene expression and mimic the effect of extra copies of X in diploid *dpy-21*(+) animals.

Subsequently, direct measurement of transcript levels determined that dosage compensation occurs in nematodes (Meyer and Casson 1986). For multiple X-linked genes, quantification of mRNA revealed the same level of transcripts in XX hermaphrodites, XX males, and XO males despite the difference in X-chromosome dose and sexual phenotype (Meyer and Casson 1986). An essential control to validate the lack of difference in transcript levels, despite the difference in X dosage, was the demonstration that the transcript level from each of these X-linked genes was directly proportional to gene copy number in XX hermaphrodites when the dose of any single gene was changed individually. One gene copy produced half the level of transcripts as two copies, and four copies produced twice the level of transcripts as two. Together, these experiments revealed the presence of an X-chromosome dosage compensation process in *C. elegans*.

Measurement of the X-linked transcripts in *dpy-21*, *dpy-27*, and *dpy-28* XX and XO mutants revealed a twofold to threefold increase in transcripts in XX but not XO *dpy* mutants (Meyer and Casson 1986). Hence, these *dpy* mutations disrupt dosage compensation and cause an elevation in X-chromosome gene expression in XX mutants.

Both the phenomenon of dosage compensation and the involvement of *dpy-21*, *dpy-26*, *dpy-27*, and *dpy-28* in X-linked gene expression were also shown by a phenotypic assay (DeLong *et al.* 1987; Meneely and Wood 1987) similar to the one originally used by Muller to demonstrate dosage compensation in fruit flies, long before molecular tools were available to study gene expression (Muller 1932). In this assay, the severity of a mutant phenotype caused by an X-linked partial loss-of-function mutation (plof) was used to indicate the level of gene activity, with increasing allele dose associated with increasingly normal phenotype. Muller found that the phenotype caused by a single X-linked plof allele in XY males (plof/Y) was identical to the phenotype caused by two plof alleles in XX females (plof/plof), despite the fact that one plof allele in XX females (plof/deletion) was more mutant than one plof allele (plof/Y) in XY males. From these results, Muller hypothesized that the total level of X expression in the male with one X chromosome must be similar to the level of expression in the female with two X chromosomes, hence a dosage compensation process must balance gene expression between XY males and XX females (Muller 1932).

In worms, as for most X-linked plofs in fruit flies, phenotypes caused by X-linked plof mutations were found to be equivalent in XX and XO animals, indicating similar levels of X-linked products in both sexes, despite the difference in number of mutant copies, and hence a dosage compensation process (DeLong *et al.* 1987; Meneely and Wood 1987). In this phenotypic assay, an increase in expression of an X-linked plof mutation would ameliorate mutant phenotypes, whereas a reduction would exacerbate them. Mutations in *dpy-21*, *dpy-26*, *dpy-27*, and *dpy-28* suppressed the mutant phenotypes caused by plof mutations in several different genes in XX but not XO animals, indicating an elevation in X-linked gene expression specifically in XX animals and hence disruption of dosage compensation (DeLong *et al.* 1987; Meneely and Wood 1987).

The XX-specific elevation in X-linked gene expression in worms was consistent with either of two mechanisms for dosage compensation: random inactivation of a single hermaphrodite X chromosome or repression of both hermaphrodite X chromosomes by half. X-inactivation was unlikely since neither of two genetic phenomena it would cause were observed. Hermaphrodites were not mosaic in phenotype when heterozygous for cell-autonomous X-linked mutant genes (m/+), and most X-linked loss-of-function mutations failed to behave as dominant alleles with variable penetrance and expressivity. Subsequent experiments presented below demonstrate that dosage compensation is achieved by reducing transcription from both hermaphrodite X chromosomes by half.

Following the identification of the original dosage-compensation *dpy* mutations, the gene *dpy-30* was found to play an essential role in dosage compensation (Hsu and Meyer 1994; Hsu *et al.* 1995). It resembled the other maternally provided dosage compensation genes in causing overexpression of X-linked genes and complete lethality in XX animals when mutant. However, *dpy-30* mutations also affected development of XO males. Approximately 20% of males were inviable, and the viable males were scrawny, developmentally delayed, and mating defective due to aberrant tail morphology (Table 1). The combined phenotypes in XX and XO animals suggested that *dpy-30* might have a more general role in *C. elegans* development, indicating the dosage compensation process might share components important to other processes in both sexes (Hsu and Meyer 1994; Hsu *et al.* 1995).

A proteomics approach also identified a component required for dosage compensation, CAPG-1 (Csankovszki *et al.* 2009). This subunit was identified by immunoprecipitation of the complex with antibodies to DPY-27 followed by analysis through mass spectroscopy. Mutations in the *capg-1* gene cause the death of XX animals.

### Coordinate control of hermaphrodite sex determination and dosage compensation

The dosage compensation *dpy* genes regulate X-chromosome gene expression in the context of other XX-specific genes that coordinately control both sex determination and dosage compensation, the *sdc* genes (Figure 2A;Table 1) (Villeneuve and Meyer 1987; Nusbaum and Meyer 1989; Villeneuve and Meyer 1990; DeLong *et al.* 1993; Klein and Meyer 1993; Dawes *et al.* 1999). *sdc-2* is the pivotal gene that activates the dosage compensation process in XX animals and also sets the hermaphrodite mode of sex determination by repressing the male-determining gene *her-1* (Nusbaum and Meyer 1989; Dawes *et al.* 1999). Null mutations in *sdc-2* have no effect on otherwise wild-type XO animals, but cause extensive lethality in XX animals, similar to mutations in the dosage-compensation *dpy* genes. *sdc-2* mutations also cause complete reversal of sexual fate in the rare escapers, resulting in severely masculinized XX animals. The effects of *sdc-2* mutations on sex determination and dosage compensation are implemented by two independent pathways, as illustrated by the fact that masculinization, but not lethality, is blocked my mutation of *her-1* (Figure 2C).

*sdc-2* acts as a hermaphrodite switch gene (Dawes *et al.* 1999). SDC-2 protein is made exclusively in XX animals and has no maternal effect. Ectopic expression of *sdc-2* transcripts in XO animals causes extensive (∼90%) XO-specific lethality that is suppressed by mutations in the XX-specific dosage-compensation *dpy* genes. Rescued XO animals develop as hermaphrodites, because the *dpy* mutations suppress the defects in X gene expression but not in sex determination. SDC-2 binds to X chromosomes to trigger dosage compensation and recruits all other dosage compensation proteins to X (Dawes *et al.* 1999).

The first *sdc* gene to be discovered was *sdc-1* (Villeneuve and Meyer 1987, 1990). It acts at the same place in the genetic hierarchy as *sdc-2* (Figure 2A;Table 1), but it is maternally rescuable, and its null phenotype is relatively weak: not all XX animals are masculinized, and the masculinization itself is incomplete. Moreover, null *sdc-1* alleles cause no significant XX-specific lethality, despite causing some overexpression of X-linked genes and a Dpy phenotype. Nonetheless, synergy occurs between alleles of *sdc-1* and *sdc-2*, demonstrating the importance of their joint participation. The combination of a weak *sdc-2* allele that causes little or no lethality by itself and a null *sdc-1* allele that is also nonlethal, results in complete XX-specific lethality and masculinization.

*sdc-2* also collaborates with *sdc-3* to achieve proper dosage compensation and sex determination (Figure 2A;Table 1). *sdc-3* differs from the other XX-specific coordinate-control genes in that its sex-determination and dosage-compensation activities are separately mutable, indicating they function independently (DeLong *et al.* 1993; Klein and Meyer 1993; Davis and Meyer 1997). Three different classes of mutant *sdc-3* alleles were identified genetically (DeLong *et al.* 1993; Klein and Meyer 1993). One class (*sdc-3* Tra) is in a putative ATP binding motif. Mutations in this class masculinize XX animals by elevating *her-1* transcript levels but have no effect on dosage compensation. A second class (*sdc-3* Dpy) is in the two zinc fingers and disrupts dosage compensation, causing more than 95% XX-specific lethality. This class has little or no effect on sex determination. The zinc fingers are essential for the localization of SDC-3 to X chromosomes (Davis and Meyer 1997). These two classes complement each other fully, as if they represented two separate genes. A third class, comprised of true null alleles, fails to complement alleles in either of the first two classes. Ironically, the null phenotype itself is misleading, since it does not reflect the gene’s involvement in sex determination: escapers are not masculinized. Extensive genetic and molecular analysis revealed that the dosage-compensation defect of *sdc-3* null alleles suppresses their sex-determination defect as a consequence of a general feedback loop between sex determination and dosage compensation. In this feedback loop, mutations that disrupt dosage compensation can ameliorate the masculinizing effects of either partial loss-of-function mutations in some genes that control hermaphrodite sexual differentiation or gain-of-function mutations in the *her-1* male-determining gene that cause partial masculinization of XX animals (DeLong *et al.* 1993).

All SDC proteins act in concert to regulate sex determination and dosage compensation (Figure 2A). They bind directly to multiple sites in the 5′ regulatory region of the male-determining gene *her-1* to repress it and thereby promote hermaphrodite sexual development (Chu *et al.* 2002). They also bind to X chromosomes to regulate X gene expression. For *her-1*, SDC-2 binding requires SDC-3 but not SDC-1. For X, the opposite is true (Dawes *et al.* 1999; Chu *et al.* 2002; Yonker and Meyer 2003). SDC-3 binding requires SDC-2, but not SDC-1 (Yonker and Meyer 2003).

### The dosage compensation machinery: a molecular motor that controls higher-order chromosome structure

**Figure 6:**
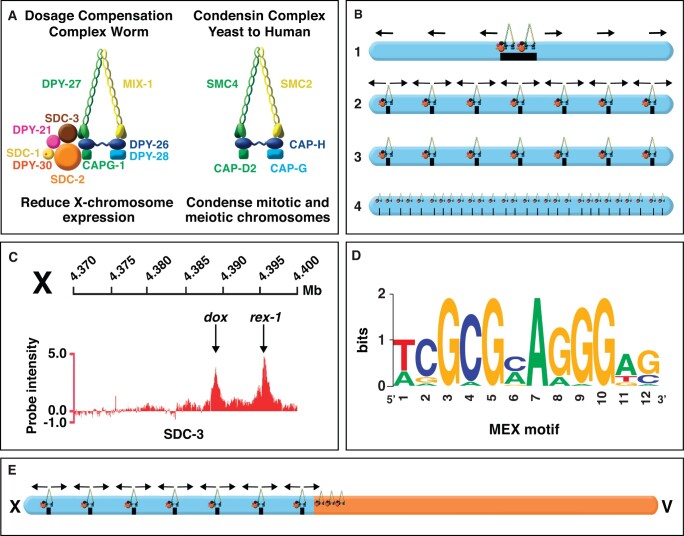
Targeting the dosage compensation complex to X chromosomes. **(**A) The DCC contains 10 identified subunits, including five condensin-like subunits (DPY-27, MIX-1, DPY-26, DPY-28, and CAPG-1) that are homologous to canonical condensin subunits SMC2, SMC4, CAP-H, CAP-D2, and CAP-G1, respectively, which are conserved from yeast to human. The DCC also includes the XX-specific novel protein SDC-2 that is expressed specifically in XX animals and triggers assembly of the DCC onto X. Two DCC subunits aid SDC-2 in recruiting the complex to X: SDC-3 (a zinc-finger protein) and DPY-30 (also a subunit of the MLL/COMPASS H3K4me3 methyltransferase complex). Two additional subunits, SDC-1 (a zinc-finger protein) and DPY-21 (Jumonji C H4K20me2 demethylase), are required for DCC activity but not for assembly of the DCC onto X. (B) Possible models for the mechanism by which the DCC is targeted to X. A single site on X could recruit the DCC and nucleate spreading across X (1). A limited number of sites could recruit the DCC and either nucleate DCC spreading (arrows) (2) or not (3). If no spreading occurs, the DCC would act over long distance to repress gene expression (3). A high density of sites could recruit the DCC but no spreading would occur, implying direct, short-range gene regulation by the DCC (4). Model 2 representing DCC recruitment to specific sites on X followed by spreading is the mechanism supported by all available data. (C) Enlargement of the DNA section from the 4.37- to 4.40-Mb region on the left end of X showing adjacent *rex* and *dox* DCC binding sites mapped by ChIP-chip (shown) and ChIP-seq experiments and assayed for autonomous DCC recruitment ability *in vivo*. Sites were classified into two categories based on their ability to bind the complex when detached from X chromosomes. *rex* sites (recruitment elements on X) bind the complex robustly *in vivo* when they are detached from X and are present either in multiple copies on extrachromosomal arrays or in low copy number integrated onto an autosome. *dox* sites (dependent on X) fail to bind the DCC when detached from X and require the X-chromosome context of *rex* sites for their DCC binding ability. DCC binding at *rex* sites facilitates binding at *dox* sites nearby, but the mechanism of spreading is not known. (D) A 12 base pair consensus motif identified by motif searches is enriched at *rex* sites relative to *dox* sites and on X chromosomes relative to autosomes. It recruits the DCC to X but cannot be the sole X-enriched motif to do so. Mutations within the motif disrupt the ability of *rex* sites to bind the DCC. (E) DCC binding to chromosome V is facilitated by proximity to *rex* sites located on the X part of an X:V fusion chromosome. DCC binding on X is able to spread into the 2 Mb region of chromosome V adjacent to the fusion break point. Chromosomes X (17.7 Mb) and V (20.9 Mb) are drawn to scale.

**Figure 7:**
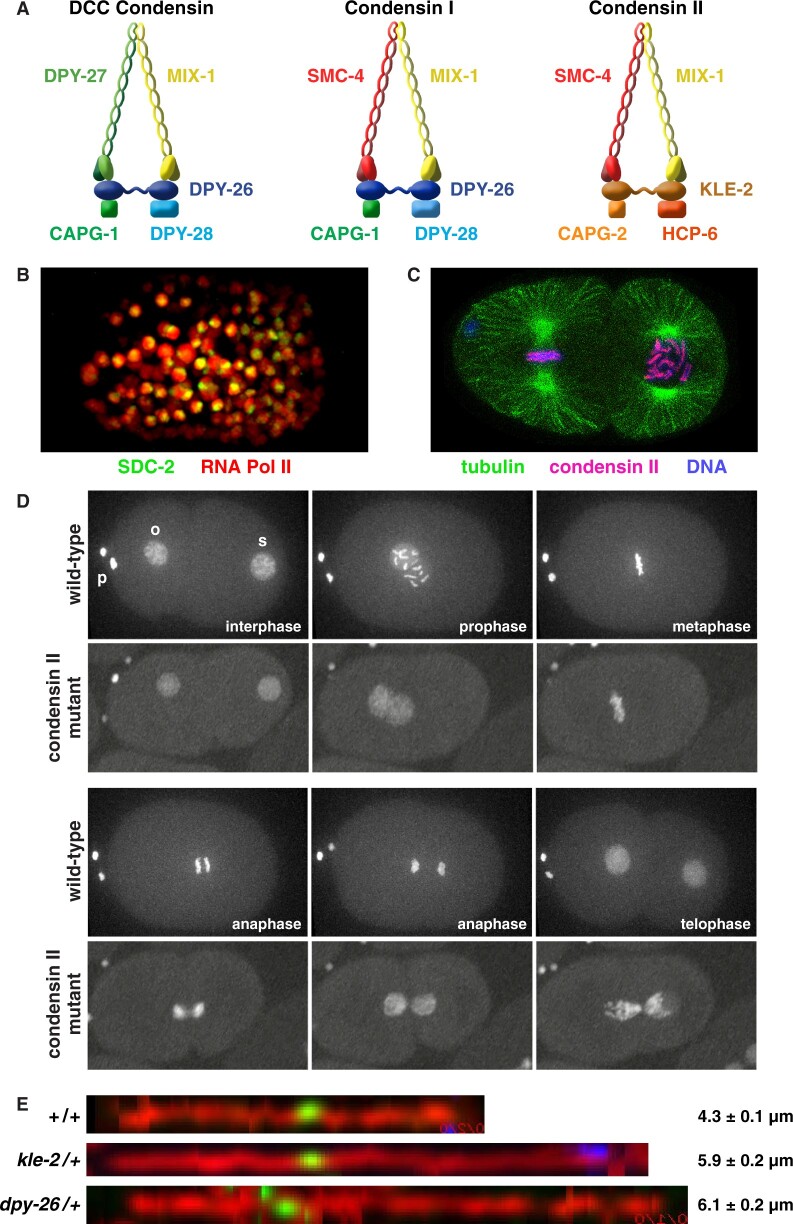
Three condensin complexes carry out distinct functions in *C. elegans.* (A) Comparison of the DCC condensin complex compared with the two other independent condensin complexes in *C. elegans*. The DCC condensin binds to X chromosomes and reduces X expression in XX embryos (B). It shares four subunits with condensin I as shown; DPY-27 replaces SMC-2 as the fifth subunit. Condensin I plays minor roles in chromosome segregation during mitosis and meiosis. Condensin II is the prime condensin complex responsible for mitotic and meiotic chromosome compaction, resolution, and segregation. It shares one subunit with the DCC (MIX-1) and two subunits (SMC-4 and MIX) with condensin I. (B) SDC-2 is bound to both X chromosomes. Shown is an XX embryo expressing SDC-2::mNeonGreen (green) and RNA Polymerase II::mRuby (red), which is dispersed throughout the nucleus. (C) Condensin II binding on holocentric mitotic chromosomes. Shown is a two-cell embryo with one cell in metaphase (left) and one in prophase (right). Condensin II (magenta) colocalizes with holocentric chromosome binding proteins all along the outer edge of each chromosome (blue), adjacent to where the mitotic spindle (green) attaches. (D) Disruption of condensin II causes severe defects in mitotic chromosome segregation. Shown is a progression of images, from fertilization through the first cell division, of a single wild-type or *hcp-6* mutant embryo carrying GFP::H2B histone-tagged chromosomes. In *hcp-6* mutants, prophase chromosomes are not properly condensed, chromosomes fail to align properly on the metaphase plate, and chromatin bridges occur between separating homologous chromosomes in anaphase, thereby preventing chromosome segregation, as seen by the fully connected sperm and oocyte chromosomes in telophase. o, oocyte pronucleus; s, sperm pronucleus; p, polar bodies. (E) Axis lengths of meiotic pachytene chromosomes are extended in mutants depleted of condensin I or condensin II. Shown are images of computationally straightened X-chromosome axes in pachytene nuclei of wild-type animals or heterozygous condensin mutants that were labeled for the cohesin axis protein COH-3/4 (red), a center X FISH probe (green), and a right end X FISH probe (blue). Chromosomes are displayed horizontally. Genotypes of animals and average total chromosome axis length with SEM are shown adjacent to each image.

In a surprising feat of evolution, regulation of X-chromosome gene expression is functionally related to a structural problem relevant to all chromosomes in dividing cells: achieving ordered compaction and resolution prior to their segregation (Figures 6A and 7A; Table 1). Five of the ten DCC subunits (MIX-1, DPY-26, DPY-27, DPY-28, and CAPG-1) resemble subunits of condensin (Figure 6A) (Chuang *et al.* 1994; Lieb *et al.*^1996^, ^1998^; Tsai *et al.* 2008; Csankovszki *et al.* 2009; Mets and Meyer 2009). Condensin is an essential complex that regulates the organization, resolution, and segregation of chromosomes during mitosis and meiosis from yeast to humans (Hirano 2016; Yatskevich *et al.* 2019). All DCC condensin subunits except DPY-27 also control the structure and function of mitotic and meiotic chromosomes in XX and XO animals by participating in other biochemically distinct condensin complexes, condensin I and condensin II (Figure 7A) (Lieb *et al.* 1998; Hagstrom *et al.* 2002; Csankovszki *et al.* 2009; Mets and Meyer 2009) (Figure 7A). The functions of condensin I in chromosome segregation are minor (Csankovszki *et al.* 2009; Mets and Meyer 2009), but the functions of condensin II are major (Figure 7, A and D) (Hagstrom *et al.* 2002), as will be discussed later. Mutations in *dpy-26* and *dpy-28* also cause X-chromosome nondisjunction, resulting in a higher incidence of XO males in a brood due to loss of an X chromosome (Hodgkin 1983; Plenefisch *et al.* 1989; Tsai *et al.* 2008). The mechanism and molecules involved in nondisjunction are not known. Thus, not only did the worm co-opt condensin subunits for a new role in regulating gene expression but also it continued to use these proteins in their ancient roles of regulating chromosome structure to achieve faithful chromosome segregation.

The DCC, like mitotic condensin, contains a pair of proteins (DPY-27 and MIX-1) that belong to the SMC (Structural Maintenance of Chromosomes) family of chromosomal ATPases (Chuang *et al.* 1994; Lieb *et al.* 1998) (Figures 6A and 7A). Each has nucleotide-binding domains (NBDs) at its N- and C-termini that are linked by two long coiled coil domains separated by a hinge domain (Figures 6A and 7A). Each SMC protein folds back on itself to form a central region of two anti-parallel coiled coils flanked by the NBDs and the hinge. DPY-27 and MIX-1 dimerize through interactions between their hinge domains and use their globular NBDs to bind the three non-SMC condensin DCC proteins (DPY-26, DPY-28, and CAPG-1). Mutation of the NBDs in DPY-27 and MIX-1 disrupts dosage compensation. MIX-1 also participates in two other complexes, condensin I and condensin II, to carry out independent roles in chromosome segregation, as will be discussed later (Figure 7, A, C, and D) (Csankovszki *et al.* 2009; Mets and Meyer, 2009).

Of the five noncondensin DCC subunits, one subunit (DPY-30) also participates in the MLL/COMPASS histone methyltransferase complex that trimethylates lysine 4 on histone H3 (H3K4me3), an essential histone modification needed for activation of gene expression genome-wide in both XX and XO animals (Pferdehirt *et al.* 2011). In contrast, DPY-30 lacks this activity when part of the DCC and represses gene expression instead. DPY-30 binds to different genomic locations depending on whether it participates in the MLL/COMPASS complex or the DCC (Pferdehirt *et al.* 2011).

Another subunit (DPY-21) is a Jumonji C H4K20me2 demethylase that catalyzes the conversion of H4K20me2 to H4K20me1 (Brejc *et al.* 2017). DPY-21 is required for the enrichment of H4K20me1 on hermaphrodite X chromosomes during dosage compensation, as will be discussed later.

All subunits are recruited to X chromosomes by DCC subunits SDC-2 and SDC-3, which coordinately control both sex determination and dosage compensation (Figure 2A) (Chuang *et al.* 1994, 1996; Hsu *et al.* 1995; Lieb *et al.* 1996, 1998; Davis and Meyer 1997; Dawes *et al.* 1999; Tsai *et al.* 2008; Csankovszki *et al.* 2009; Pferdehirt *et al.* 2011; Brejc *et al.* 2017). SDC-2 is the sole dosage compensation protein expressed exclusively in hermaphrodites, and it triggers assembly of the DCC onto X chromosomes in young XX embryos around the 30- to 40-cell stage (Figure 7B) (Dawes *et al.* 1999). It achieves both sex-specificity and X-specificity for the dosage compensation process (Dawes *et al.* 1999). SDC-3, a zinc-finger protein, assists SDC-2 in the X-recruitment process, as does DPY-30 (Figure 2A) (Klein and Meyer 1993; Hsu *et al.* 1995; Davis and Meyer 1997; Dawes *et al.* 1999; Pferdehirt *et al.* 2011). Mutation of *sdc-2*, *sdc-3*, or *dpy-30* prevents all DCC condensin subunits from binding to X. The final subunit, SDC-1, a zinc-finger protein, is required for the repressive activity of the DCC, but not for its recruitment to X (Nonet and Meyer 1991; Chuang *et al.* 1996; Chu *et al.* 2002). A hierarchy of dependence occurs for binding of DCC subunits to X. All DCC subunits require SDC-2 for their binding to X, but SDC-2 can bind X without other DCC subunits (Chuang *et al.* 1994, 1996; Hsu *et al.* 1995; Lieb *et al.* 1996, 1998; Davis and Meyer 1997; Dawes *et al.* 1999; Chu *et al.* 2002; Yonker and Meyer 2003; Tsai *et al.* 2008; Pferdehirt *et al.* 2011). DPY-30 requires only SDC-2 for its assembly on X, and SDC-3 requires both SDC-2 and DPY-30 (Hsu *et al.* 1995; Davis and Meyer 1997; Pferdehirt *et al.* 2011). All condensin subunits and both SDC-1 and DPY-21 subunits also require SDC-2, DPY-30, and SDC-3 for their full association with X.

Post-translational modification by the SUMO (small ubiquitin-like modifier) conjugation pathway is essential for sex-specific assembly of the DCC on X (Pferdehirt and Meyer 2013). SDC-3 and condensin subunits DPY-27 and DPY-28 are SUMOylated *in vivo* and depletion of SUMO *in vivo* severely disrupts binding of these subunits to X, causing overexpression of X-linked genes.

### Participation of dosage compensation proteins in mitotic and meiotic chromosome segregation

The condensin subunits of the DCC also participate in other complexes that carry out independent roles in chromosome segregation (Figure 7, A, C, and D) (Lieb *et al.* 1998; Hagstrom *et al.* 2002; Chan *et al.* 2004; Tsai *et al.* 2008; Csankovszki *et al.* 2009; Mets and Meyer 2009; Chao *et al.* 2017). The SMC subunit MIX-1 participates in both condensin I and condensin II (Figure 7A) (Lieb *et al.* 1998; Csankovszki *et al.* 2009). Condensin I differs from the DCC condensin by only one subunit: SMC-4 replaces its SMC paralog DPY-27 (Figure 7A) (Csankovszki *et al.* 2009; Mets and Meyer 2009). Condensin II includes both MIX-1 and SMC-4 but differs from both the DCC and condensin I in having non-SMC proteins that are distinct from, but homologous to, those of the other two complexes (KLE-2, CAPG-2, and HCP-6) (Figure 7A) (Csankovszki *et al.* 2009; Mets and Meyer 2009). Condensin II is the primary condensin complex in *C. elegans* to control the compaction and resolution of mitotic and meiotic chromosomes in preparation for their segregation (Figure 7, D and E) (Lieb *et al.* 1998; Hagstrom *et al.* 2002; Csankovszki *et al.* 2009; Mets and Meyer 2009). The participation of dosage compensation proteins in diverse condensin complexes illustrates that reshuffling of homologous, interchangeable molecular parts can create independent machines with similar architecture but distinct cellular localization and biological functions.

During mitosis, condensin II subunits colocalize with holocentric proteins at the outer edge of chromosomes where the spindle attaches (Figure 7C) (Lieb *et al.* 1998; Hagstrom *et al.* 2002). Condensin II depletion disrupts mitotic prophase condensation (Figure 7D), holocentromere organization, and chromosome segregation (Figure 7D) (Lieb *et al.* 1998; Hagstrom *et al.* 2002; Csankovszki *et al.* 2009). Chromosome segregation defects are severe and result in the death of both XX and XO embryos.

During meiosis, condensin II acts as a chromosome-restructuring complex to drive the transformation of homologous chromosomes from their extended, parallel arrangement in pachytene nuclei to the compact cruciform structure of sister chromatids in diakinesis (Hagstrom *et al.* 2002; Chan *et al.* 2004). Condensin II depletion prevents chromosome segregation during both the first and second meiotic divisions, thereby blocking extrusion of both polar bodies and causing aneuploidy (Hagstrom *et al.* 2002; Chan *et al.* 2004; Csankovszki *et al.* 2009). Depletion of condensin II subunits also extends the axes of pachytene chromosomes (Figure 7E) (T. W. Lee and B. J. Meyer, unpublished; Lee 2014).

Condensin I associates with condensed mitotic chromosomes of XX and XO embryos in a discontinuous pattern (Csankovszki *et al.* 2009). Condensin I depletion causes chromatin bridges between anaphase chromosomes, resulting in a mild-mitotic chromosome segregation defect (Csankovszki *et al.* 2009; Hernandez *et al.* 2018) that is less severe than that caused by condensin II depletion (Hagstrom *et al.* 2002; Csankovszki *et al.* 2009). Most XO embryos depleted of condensin I were viable, and animals survive to adulthood.

Condensin I surrounds meiotic chromosomes in prophase and then localizes to regions between the paired homologous chromosomes in the meiosis I division and the paired sister chromatids in the meiosis II division (Csankovszki *et al.* 2009; Hernandez *et al.* 2018). Condensin I depletion causes mild defects in homolog pairing, in sister chromatid cohesion, and in chromosome segregation (Csankovszki *et al.* 2009; Hernandez *et al.* 2018). Condensin I depletion also causes extension of pachytene chromosome axes (Figure 7E) and a dominant change in the distribution of RAD-51-marked DNA double-strand-break-dependent recombination intermediates and crossovers between homologous chromosomes (Mets and Meyer 2009).

### Targeting the DCC to X chromosomes: *cis*-acting sites on X that recruit the DCC in somatic cells

In somatic cells, all DCC condensin and SDC subunits bind to X chromosomes starting around the 30- to 40-cell stage of embryogenesis (Figure 7B), and DCC binding is maintained through adulthood (Dawes *et al.* 1999; Pferdehirt *et al.* 2011). The onset of dosage compensation in the embryo is linked to the loss of pluripotency and start of cellular differentiation (Custer *et al.* 2014). A key challenge has been to identify the features of X that recruit the DCC. In initial studies to identify *cis*-acting X-chromosome recruitment sites, a chromosome-wide search was conducted to define megabase-size regions of X sufficient to recruit the DCC when detached from X (Csankovszki *et al.* 2004). Regions were analyzed in 32-ploid intestinal cell nuclei of XX hermaphrodite strains carrying either free or autosome-attached X-chromosome duplications. If X chromosomes contained discrete DCC recruitment elements, four main scenarios were plausible for how the DCC might be targeted to X (Figure 6B). First, a single site on X could recruit the complex and nucleate long-range DCC spreading across the entire X. Second, a limited number of sites could recruit the complex, and some or all sites could nucleate short-range spreading. Third, a limited number of sites could recruit the complex but the complex would not spread, suggesting the complex would influence gene expression from long distance, perhaps by controlling chromosome structure. Fourth, a high density of sites could recruit the complex but no spreading would occur, implying direct, short-range regulation by the complex.

The experiments revealed that many detached, nonoverlapping X regions recruited the complex, indicating multiple independent recruitment sites (Csankovszki *et al.* 2004). Also, X regions were found that did not recruit the complex when detached, implying a limited number of sites rather than a high density of sites. However, regions lacking binding when detached from X had abundant DCC binding on native X chromosomes and harbored well-defined dosage-compensated genes on X. Thus, the X chromosome has discrete X recruitment sites that must nucleate DCC spreading (model 2, Figure 6B) (Csankovszki *et al.* 2004).

Two approaches then defined DCC recruitment sites (<1 kb) within these larger recruitment regions. First, a random set of cosmids from three different 2-Mb recruiting regions of X (Csankovszki *et al.* 2004; McDonel *et al.* 2006) were introduced into worms and assayed for their ability to recruit the DCC *in vivo*, as were all cosmids from another 2-Mb recruiting region of X (Jans *et al.* 2009). DCC recruitment was assessed by immunofluorescence experiments using DCC antibodies and cosmid DNA FISH probes to quantify whether the DCC bound to extra-chromosomal arrays carrying multiple copies of individual cosmids in transgenic animals. Recruitment was then ascribed to successively smaller DNA fragments using the array assays, thereby defining 17 recruitment elements on X (*rex*) sites. Second, DCC-binding sites were identified without regard to recruitment ability through a series of biologically independent chromatin-immunoprecipitation (ChIP) experiments to different DCC components, including SDC-2, SDC-3, DPY-27, and MIX-1. The precipitated DNA was hybridized to genome-wide high-resolution tiling arrays to identify DCC binding sites (Ercan *et al.* 2007; Jans *et al.* 2009; Pferdehirt *et al.* 2011). From 63 of the strong ChIP-chip peaks identified across X and assayed for recruitment ability *in vivo* using the extra-chromosomal assay described above, 14 additional *rex* sites were identified (Jans *et al.* 2009). The DCC-binding peaks identified on the endogenous X that did not exhibit autonomous DCC binding when detached from X in recruitment assays were classified as dependent on X (*dox*) sites (Figure 6C). Even though the DCC spreads across X to *dox* sites, its binding remains the highest on *rex* sites.

Sequence analysis of the 31 *rex* sites revealed a robust 12-base-pair motif (Figure 6D) that is highly enriched on X chromosomes compared with autosomes and is called Motif Enriched on X (MEX) (Jans *et al.* 2009). The MEX motif is an extended version of a 7-bp motif that was found in the four original *rex* sites and was tested for functional importance by mutagenesis (McDonel *et al.* 2006) and of a 10-bp version from the largest DCC ChIP-chip peaks (Ercan *et al.* 2007). Mutational analysis *in vivo* using the array assays established the functional importance of the additional base pairs in the 12-bp motif (Jans *et al.* 2009). *rex* sites have the 12-bp motif with varying matches to the consensus sequence and hence varying degrees of enrichment on X, ranging from 4- to 25-fold. The stronger the match to the consensus sequence, the greater the enrichment on X. Most sites have multiple motifs, albeit several with lower-end motif consensus matches, consistent with the importance of motif clustering in DCC recruitment (McDonel *et al.* 2006; Jans *et al.* 2009; Pferdehirt *et al.* 2011; Albritton *et al.* 2017). Some *rex* sites lack high-scoring MEX motifs (Pferdehirt *et al.* 2011). The MEX motif is widely distributed on X; it is coincident with DCC peaks defined by the ChIP-chip and ChIP-seq experiments; and the motif variants on X with highest enrichment on X are predictive of *rex* sites, as shown by recruitment array assays performed i*n vivo* (Pferdehirt *et al.* 2011). However, the high-scoring MEX motifs are only predictive of *rex* sites provided that binding of DCC condensin subunits at these motifs is eliminated by *sdc-2* mutations, and the regions adjacent to the high-scoring MEX motifs lack the active histone modification H3K4me3 (Pferdehirt *et al.* 2011). This modified histone correlates with high levels of transcription, a feature that is not common to *rex* sites.

The MEX motif cannot be the sole basis for conferring X specificity to DCC binding. Only 70% of the *rex* sites have a high-scoring MEX motif that overlaps with high DCC-occupancy at a *rex* site (Jans *et al.* 2009). Moreover, a few strong MEX motifs occur on autosomes but do not coincide with strong DCC binding peaks, and not even all high-occupancy DCC binding sites on X have a high-scoring MEX motif (Jans *et al.* 2009; Pferdehirt *et al.* 2011; Albritton *et al.* 2017).

### Recruitment and spreading of the DCC along X

A DCC recruitment and spreading model for distributing the DCC along X requires the occurrence of two classes of binding sites on X with different DCC recruitment abilities: *rex* sites that recruit the DCC when detached from X and *dox* (dependent on X) sites that bind the DCC only when adjacent to a *rex* site or attached to an intact X (Figure 6C) (Jans *et al.* 2009). To determine whether some DCC peaks had the properties of *dox* sites, DCC peaks that were adjacent to four different *rex* sites and similar in size to the *rex* sites were tested in the recruitment assay. Strong peaks ranging from 2 to 6 kb away from *rex* sites failed to recruit the DCC in the array assay, consistent with a model of DCC targeting involving DCC recruitment to specific *rex* sites and DCC binding to adjacent sites in a nonautonomous manner. The generality of the results was confirmed by systematically assaying recruitment ability of DNA corresponding to all peaks in two 190-kb intervals (Jans *et al.* 2009). Only two peaks in each interval from a total of 30 assayed peaks had any recruitment ability. The lack of autonomous DCC binding *in vivo* to the DNA within peaks confirmed the existence of *dox* sites. *dox* sites are more prevalent than *rex* sites. *rex* and *dox* sites are interspersed and can be separated by long distances (2–90 kb). DCC binding along X is highly nonuniform. The actual number of *rex* sites is not known but estimates suggest at least 100 *rex* sites on X (Jans *et al.* 2009; Pferdehirt *et al.* 2011).

Other features distinguish *rex* sites from *dox* sites. *dox* sites lack variants of the MEX motif that have the highest enrichment on X and are present in the strongest *rex* sites (Jans *et al.* 2009). Furthermore, *dox* sites, unlike *rex* sites are found preferentially in expressed genes and are biased toward promoters, with highly expressed genes having the majority of *dox* sites (Pferdehirt *et al.* 2011). Moreover, the pattern of DCC binding at *dox* sites changes dynamically throughout development as genes are turned on and off, with a positive correlation between the level of gene expression and the level of DCC binding. In contrast, minimal, if any, changes in DCC binding occur at *rex* sites in response to changes in gene expression (Pferdehirt *et al.* 2011).

Binding of DCC condensin subunits at *rex* sites has a nearly absolute requirement for SDC-2, SDC-3, and DPY-30 (Pferdehirt *et al.* 2011). Mutations in any of these genes eliminates *rex* binding of condensin subunits. In contrast, SDC-3 binding at *rex* sites does not require condensin subunits but does require both SDC-2 and DPY-30. Binding of DPY-30 at *rex* sites with other DCC subunits requires SDC-2 but not SDC-3. Finally, SDC-2 binding at *rex* sites occurs independently of the other subunits; SDC-2 binding can occur without SDC-3, DPY-30, or condensin subunits. Hence, SDC-3, DPY-30, and condensin subunits are unlikely to play significant roles in sequence-specific recognition of *rex* sites (Pferdehirt *et al.* 2011).

DCC binding at *dox* sites is more complex. Maximal binding of condensin subunits at all *dox* sites requires SDC-2, SDC-3, and DPY-30, but binding at many *dox* sites can occur at reduced levels in an SDC-independent and DPY-30-independent manner (Pferdehirt *et al.* 2011). That is, some *dox* sites have an inherent, low-level ability to bind condensin subunits independently of the genetic hierarchy that governs sex-specific DCC loading onto X. This low-level of SDC-independent condensin binding likely reflects the general binding properties of mitotic condensin in interphase chromosomes. The mitotic condensin-specific subunit SMC-4, a paralog of the DCC-specific DPY-27 protein, has a profile of X binding in wild-type embryos that closely resembles the pattern for DPY-27 in *sdc-2* mutant embryos. Of *dox* sites that have SMC-4 binding in wild-type embryos, 90% retain DPY-27 binding in *sdc-2* null mutants (Pferdehirt *et al.* 2011). In wild-type embryos, the level of DCC condensin binding at many *dox* sites is greater than the level of SDC binding, implying that while SDC proteins are essential for maximal DCC condensin binding at *dox* sites, a simple one-to-one ratio between SDC subunits and condensin subunits is not essential at *dox* sites for full gene repression (Pferdehirt *et al.* 2011; Albritton *et al.* 2017). Furthermore, spreading of DCC condensin to *dox* sites may occur without the concomitant spreading of SDC subunits to these sites.

To further understand the relationship between *rex* sites and *dox* sites, DCC binding was assessed in one study after high-occupancy *rex* sites were either deleted from the endogenous X chromosome or high-occupancy *rex* sites were inserted into new locations on the wild-type X or an X with *rex* deletions (Anderson *et al.* 2019). Eight of the highest-occupancy *rex* sites across X, which also drive higher-order X-chromosome structure (discussed later), were deleted sequentially from the endogenous X, and the effects on binding of condensin subunit DPY-27 and DCC loader SDC-3 were evaluated by ChIP-seq. Binding of the two DCC subunits was reduced up to 16-fold immediately adjacent to the *rex* deletions and gradually returned to wild-type levels at approximately 20 kb on either side of each deleted site (Anderson *et al.* 2019). Reciprocally, when strong *rex* sites were inserted into new locations on wild-type X chromosomes or X chromosomes bearing deletions of the eight *rex* sites, DCC binding was enriched approximately 16-fold around the *rex* insertion sites, and binding gradually decreased to wild-type levels about 15 kb from the insertions (Anderson *et al.* 2019). Binding at *rex* sites in the new locations was equivalent in level to the binding at their endogenous locations on X. These results further support the model of DCC binding at autonomous recruitment sites and spreading to nonautonomous sites (Anderson *et al.* 2019).

When all eight *rex* sites were deleted across the entire X, DCC binding was preserved on the remaining *rex* sites, and the general change in DCC binding across the length of X was minimal. The impact on DCC binding of eliminating either individual *rex* sites or multiple sites across X was restricted to the regions near *rex* sites, implying that binding at *dox* sites is influenced locally by high-affinity DCC binding nearby (Anderson *et al.* 2019).

A separate study deleted a single *rex* site from either the left, right, or middle region of X (Albritton *et al.* 2017). In each case, the DCC binding profile on X was largely unchanged as measured by ChIP-seq, in agreement with Anderson *et al.* (2019). Albritton *et al.* (2017) did report, however, a small decrease (10–20%) in DCC binding within a 1–2 Mb region surrounding each deletion, suggesting the possibility of a small but longer-range effect on DCC binding caused by a *rex* deletion.

### Targeting the DCC to autosomes

Binding of condensin DCC subunits on autosomes in wild-type embryos resembles the residual binding of these condensin subunits to *dox* sites on X chromosomes in *sdc-2*, *sdc-3*, or *dpy-30* mutants, in both the density of bound sites and the level of occupancy at sites (Pferdehirt *et al.* 2011). By inserting *rex* sites onto autosomes, one can learn principles governing DCC binding and spreading along X.

Inserting a single *rex* site with clustered motifs onto an autosome elicited virtually no DCC binding, while inserting three high-occupancy *rex* sites with multiple clustered motifs from the center of X onto an autosome, with the same spacing intervals of 1.4 and 1.6 Mb as on X, recruited the DCC, but with only 20% of the binding at each endogenous or ectopic site on X (Anderson *et al.* 2019). These results suggests that full DCC binding at *rex* sites requires the cooperation among multiple recruitment sites or among a high density of clustered MEX motifs, as found naturally on X. This interpretation was supported by a separate study showing that one copy of a *rex* site failed to recruit the DCC to an autosome, but flanking it with copies of a different *rex* site, either 30 or 50 kb away, increased DCC binding at the first site to 30% of the endogenous level (Albritton *et al.* 2017).

The concept of DCC binding at *rex* sites being enhanced by cooperation among *rex* sites was foreshadowed by earlier studies demonstrating that strong *rex–rex* interaction frequencies are directly correlated with the level of DCC occupancy at *rex* sites (Crane *et al.* 2015). The positive correlation was shown directly by comparing DCC occupancy measurements at *rex* sites from ChIP-seq experiments to measurements of *rex–rex* interaction frequencies in wild-type and *sdc-2* mutant embryos using genome-wide chromosome conformation capture analysis. *rex–rex* interactions were found to be the most prominent interactions on X. In wild-type embryos, *rex* interaction frequencies for pairwise combinations of the 25 highest-occupancy *rex* sites across X greatly exceeded interactions for all other *rex* sites (Crane *et al.* 2015). These interactions were eliminated in *sdc-2* mutants. While these observations are key to understanding dosage compensation, the mechanisms underlying the cooperation among *rex* sites and the mechanisms underlying DCC spreading to secondary sites are as yet not known and remain an area of active exploration.

### DCC binding to autosomal sites is enhanced by proximity to *rex* sites inserted in *cis*

Further understanding of the influence of ectopic *rex* sites on DCC spreading was achieved by analyzing genome-wide binding of condensin DCC subunit DPY-27 in a strain carrying a fusion of chromosome X attached to chromosome V (Pferdehirt *et al.* 2011) (Figure 6E). The fusion enhanced DCC binding over the 2 Mb autosomal region most proximal to the fusion breakpoint. Binding decreased progressively as distance from the breakpoint increased. Two types of DCC binding were found on the V portion of the fusion chromosome: enhanced binding at sites bound with low occupancy by the DCC on the wild-type chromosome V, and new sites of binding (Pferdehirt *et al.* 2011). The new sites occurred preferentially in the promoters of active genes, as is typical for both *dox* sites and autosomal sites. Most sites showed a low level of binding by visual inspection, but the level of binding was below detection by peak-calling programs. In contrast, DCC binding along X in the X:V fusion chromosome appeared unchanged from that on the wild-type X.

The increase in DCC binding to autosomal territories located on X-to-autosome fusion chromosomes was also seen in a separate study (Ercan *et al* 2009). In this study, DCC binding to wild-type autosomes seemed negligible, and DCC binding to autosomal territories adjacent to X was interpreted as the establishment of new DCC-binding sites. However, comparisons of DCC binding to autosomes in the data sets from the two laboratories support the results that minimal DCC binding occurs even on wild-type autosomes, and the enhanced binding on autosomes is predominantly at sites of low-occupancy binding on wild-type autosomes (Pferdehirt *et al.* 2011).

Thus, DCC binding at autosomal sites can be enhanced by the proximity of *rex* sites attached in *cis*, consistent with the model that cooperative DCC binding to multiple *rex* sites enhances X-chromosome specificity to dosage compensation and facilitates DCC binding along X at *dox* sites rather than binding to autosomal sites (Pferdehirt *et al.* 2011; Albritton *et al.* 2017). This model accounts for the preference in DCC binding to X *vs* autosomes.

These results raise the question of whether the enhanced binding of DCC subunits on autosomes caused by the insertion of ectopic *rex* sites in *cis* affects gene expression. In L1 larvae, but not embryos, a small but significant decrease in gene expression was observed on the side of the autosome attached to the X chromosome (Street *et al.* 2019). Also observed on the attached autosomes was a reduction in histone modifications associated with active gene expression, as was found on endogenous X undergoing dosage compensation (Street *et al.* 2019). The reduction in both gene expression and active histone modifications was proportional to level of DCC spreading, suggesting that DCC spreading results in changes in gene expression.

### Evolution of DCC binding on X

The presence of genome-wide low-occupancy DCC condensin binding sites near promoters and *rex* sites in the absence of SDC proteins, coupled with the fact that four of five DCC subunits function in mitotic condensin, has implications for the evolutionary origins of the DCC. Low-level, nonsex-specific binding of mitotic condensin at gene promoters might have preceded the evolution of sex chromosomes and dosage compensation. Indeed, the mitotic/meiotic-specific condensin subunit SMC-4, a paralog of the dosage-compensation-specific protein DPY-27, has a profile of binding on all interphase chromosomes that closely resembles the pattern for DPY-27 binding in an *sdc-2* mutant (Pferdehirt *et al.* 2011). Through the creation of a DCC-specific condensin subunit (DPY-27) and a protein like SDC-2 that recruits the DCC to X in a sex-specific manner, mitotic condensin subunits could have been co-opted by the dosage compensation process for sex-specific, sequence-dependent binding on X to regulate gene expression over long distances by altering chromosome structure.

### Evidence that the DCC controls recruitment of RNA polymerase II to promoters to reduce gene expression

**Figure 8:**
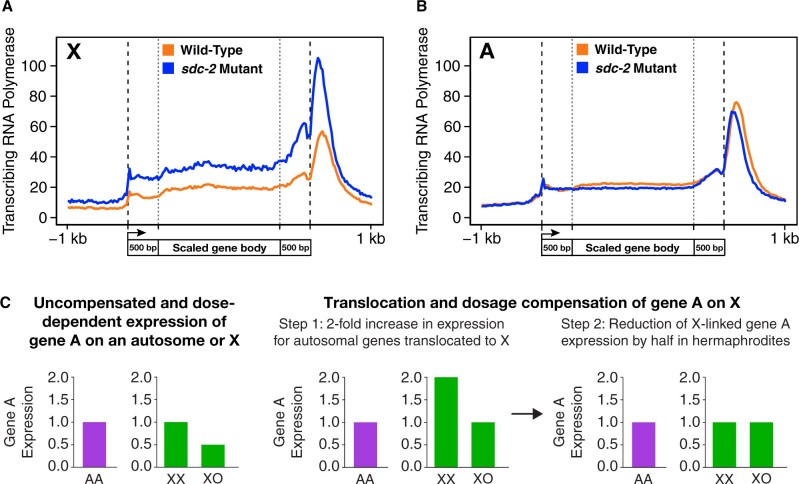
Plausible strategy for dosage compensation and for balancing gene expression between X chromosomes and autosomes. (A) Evidence that the DCC controls gene expression by limiting RNA polymerase recruitment to promoters. A uniform increase in transcriptionally engaged RNA polymerase (1.7-fold) across the length of X-linked genes, from promoters to 3′ ends, in response to disruption of dosage compensation implicates reduction of RNA polymerase recruitment to X-linked promoters as a plausible mechanism of dosage compensation. Levels of transcriptionally engaged RNA polymerase were measured by global run-on sequencing experiments. The figure shows metagene analysis comparing levels of transcriptionally engaged RNA polymerase from wild-type control embryos and *sdc-2* mutant embryos. All genes are depicted by the convention that 5′ ends (−1 kb to + 500 bp of the transcript start sites) and 3′ ends (500 bp upstream to 1 kb downstream of the 3′ end) are not scaled but all gene bodies are scaled to 2 kb. (B) Levels of transcriptionally engaged RNA polymerase on genes of autosomes are slightly decreased in *sdc-2* mutant *vs* wild-type control embryos, potentially because the limited amount of RNA polymerase in the cell is recruited to the numerous nondosage-compensated X-linked genes in the *sdc-2* mutant. Analysis was conducted and depicted as in (A). Average levels of transcriptionally engaged polymerase are similar between X and autosomal genes (Kruesi *et al.* 2013). (B) Balancing gene expression between X chromosomes and autosomes. Recognizing that the reduction of X-chromosome gene expression in XX females (or hermaphrodites) as a mechanism for dosage compensation between sexes might create a deleterious reduction in X-chromosome products for both sexes, Susumo Ohno proposed a two-step mechanism for the recruitment of autosomal genes to X chromosomes and the concomitant regulation of X-linked gene expression (Ohno 1967). During the evolution of X chromosomes from autosomes and the connected establishment of X-chromosome dosage compensation, a mechanism would arise to increase the expression level of autosomal genes translocating to X by twofold in both sexes (step 1). This upregulation of X expression would make expression from the male X equal to that of the ancestral autosomes but would cause a twofold overexpression of X-linked genes in females (or hermaphrodites) relative to the ancestral autosomes. The overexpression in females (or hermaphrodites) would then be offset by an X-chromosome dosage compensation process that reduced X expression in females (or hermaphrodites), thereby balancing X expression between sexes, as well as balancing expression between female (or hermaphrodite) X chromosomes and the ancestral autosomes (step 2). Evidence from gene expression studies supports this model for *C. elegans*.

In principle, the DCC could regulate one or more steps of transcription: recruitment of RNA polymerase II (Pol II) to promoters, initiation of transcription, escape of Pol II from promoters or pause sites, elongation of transcription, or termination of transcription. Historically, attempts to address these alternatives were thwarted by incorrect annotation of transcription start sites (TSS). Nascent RNA transcripts from most worm genes undergo rapid cotranscriptional RNA splicing in which the 5′ end is replaced by a common 22-nucleotide leader RNA, obscuring the identity of TSSs and promoters. A general strategy was devised to map TSSs, and a large TSS data set was created (Kruesi *et al.* 2013). Comparison was then made between the genome-wide distribution, orientation, and quantity of transcriptionally engaged RNA Polymerase II relative to TSSs on X and autosomes in wild-type and dosage-compensation-defective mutant XX animals using global run-on sequencing (GRO-seq) (Kruesi *et al.* 2013) (Figure 8, A and B). Promoter-proximal Pol II pausing, in which transcriptionally engaged Pol II and its attached 20–60 nucleotide-long nascent RNA accumulates downstream of promoters, occurred rarely in wild-type *C. elegans* embryos, unlike in most metazoans, and was not changed in *sdc-2* XX mutants. Thus, increasing promoter pausing cannot be the mechanism of dosage compensation. Instead, transcriptionally engaged Pol II was found to be uniformly elevated (∼1.7-fold) from promoters to 3′ ends of protein-coding genes on X in wild-type *vs* DCC mutant embryos (Figure 8A), indicating that either Pol II recruitment or Pol II initiation is the step of gene regulation controlled by the dosage compensation process, and regulation of elongation does not contribute significantly (Kruesi *et al.* 2013). Similar results were also found for microRNAs but not tRNAs, indicating that microRNAs are dosage compensated but tRNAs are not (Kruesi *et al.* 2013). Levels of transcriptionally engaged RNA polymerase on autosomal genes was decreased slightly in *sdc-2* mutants, plausibly because the limited amount of RNA polymerase in the cells was recruited to the numerous genes that fail to be dosage compensated in the *sdc-2* mutants (Figure 8B). Supporting the interpretation that Pol II recruitment or initiation is regulated by the dosage compensation process was the finding that the level of transcribing Pol II in the 5′ quarter region of each gene *vs* the distal three-quarter region was not significantly different for genes on X and autosomes in DCC-mutant *vs* control embryos. (Kruesi *et al.* 2013).

The mechanism of dosage compensation was further refined by genome-wide analysis in wild-type *vs* DCC-defective embryos of promoter-bound, nontranscribing hypo-phosphorylated Pol II *vs* the phosphorylated forms of Pol II that are indicative of either initiating or elongating Pol II (Pferdehirt *et al.* 2011). If the hypo-phosphorylated forms and phosphorylated forms are similar to each other and are uniformly elevated across the genes in *sdc-2* mutant embryos compared with wild-type embryos, then Pol II recruitment to promoters would be the likely step of transcription controlled by the DCC to repress X expression. Indeed, the level of promoter bound, nontranscribing polymerase was elevated at genes of DCC-mutant embryos *vs* wild-type embryos to the same degree as the transcribing forms of polymerase. Furthermore, the relative ratios of promoter-bound, initiating, and elongating forms of Pol II on X were uniformly about twofold higher in *sdc-2* mutants than in wild-type embryos. These results strongly support the view that the DCC controls Pol II recruitment to promoters (Pferdehirt *et al.* 2011). A definitive conclusion about the step of transcription affected by dosage compensation awaits ongoing single-molecule experiments to examine Pol II dynamics on X and autosomes in wild-type and dosage compensation mutants.

### Balancing gene expression between X chromosomes and autosomes

In organisms that equalize X-chromosome gene expression between sexes by reducing expression in XX animals, the question arises as to whether the compensated level of X expression is equivalent to or half of the expression from two sets of autosomes (Ohno 1967). The answer to this question has been controversial, although evidence has mounted in mammals in favor of a mechanism that makes total expression between X chromosomes and autosomes equal (Xiong *et al.* 2010; Deng *et al.* 2011, 2013; Disteche 2012; Jue *et al.* 2013; Larsson *et al.* 2019).

For *C. elegans*, genome-wide measurement of nascent transcripts prior to cotranscriptional processing showed that in wild-type embryos, X and autosomes had nearly equivalent levels of total gene expression and that overall levels of transcribing Pol II were uniformly equivalent across X and autosomal genes (Kruesi *et al.* 2013) (Figure 8, A and B). In dosage-compensation-defective mutants, the level of X expression and engaged Pol II exceeded that of autosomes by 1.7-fold, from the TSSs to the 3′ ends (Figure 8, A and B). These results suggested that some mechanism elevated the intrinsic rate of transcription from the X chromosomes of both sexes, so that after dosage compensation, X chromosomes and the two sets of autosomes have equivalent expression (Kruesi *et al.* 2013). From these results alone, two mechanisms are plausible in principle. The first would be to invoke a separate but ongoing chromosome-wide mechanism that increases by twofold the level of Pol II recruitment to X promoters in embryos of both sexes, prior to the enactment of dosage compensation. The second would be an evolutionary process during the formation of sex chromosomes from autosomes in which genes recruited from autosomes to X would have elevated the transcription potential of their promoters destined for X to accommodate the reduction of expression in hermaphrodites during dosage compensation on X (Figure 8C).

The first mechanism seems unlikely. Although reporter transgenes containing non-nematode genes such as *gfp* and *lacZ* driven by either X-linked or autosomal promoters became dosage compensated when integrated randomly at multiple dispersed locations along X, the per-copy transcript expression of the same transgenes integrated onto autosomes was half, not equivalent to, the average per-copy transcript expression of endogenous autosomal genes (Wheeler *et al.* 2016). If an ongoing chromosome-wide transcription process elevated expression of all genes on X in preparation for dosage compensation, then expression of the compensated transgenes on X would be equivalent to the average level of autosomal gene expression (Wheeler *et al.* 2016). The lack of equivalent expression suggests that an evolutionary process occurred to increase transcription potential for autosomal genes destined for X (Figure 8C).

### Condensin-driven remodeling of X-chromosome topology during dosage compensation: the DCC acts at a distance to regulate gene expression

Interphase chromosomes are organized into a series of ordered structures ranging from kilobase-scale chromatin loops that join promoters of genes with distant DNA regulatory sequences to multimegabase-scale subchromosomal territories (Bickmore and van Steensel 2013; Mirny *et al.* 2019; Ghosh and Meyer 2021). Intermediate-sized structures of about 1 megabase also occur during interphase. These structures, called topologically associating domains (TADs), are a common feature of mammalian chromosomes and confer the property that loci in one TAD interact predominantly with each other, while being insulated from interactions with loci in neighboring TADs (Dixon *et al.* 2012; Nora *et al.* 2012, 2013). This insulating property permits the action of distant DNA regulatory regions to be restricted to genes within a TAD, thereby preventing, for example, inappropriate activation of oncogenes or genes involved in pattern formation of the body (Flavahan *et al.* 2016; Hnisz *et al.* 2016; Valton and Dekker 2016; Beagan and Phillips-Cremins 2020). Mechanisms that define TAD boundaries and the biological functions of TADs have been elusive and controversial (Lupianez *et al.* 2015; Williamson *et al.* 2019; Beagan and Phillips-Cremins 2020), and *C. elegans* dosage compensation provided an excellent opportunity to explore them.

The potential value of investigating X chromosome structure and TAD formation was indicated by observations suggesting that the nematode dosage compensation process functions on a chromosome-wide basis, rather than on a gene-by-gene basis, to repress gene expression in hermaphrodites. These observations suggested that the DCC might alter the topology of interphase X chromosomes to regulate X expression. First, the DCC acts at a distance on the endogenous X chromosome to repress transcription across the entire X chromosome. Stable DCC binding near an endogenous X-linked gene is neither necessary nor sufficient for the dosage compensation of that gene (Jans *et al.* 2009). Second, reporter transgenes carrying non-nematode genes such as *gfp* and *lacZ* driven either by X-linked or autosomal gene promoters became dosage compensated when integrated randomly at multiple dispersed locations along X (Wheeler *et al.* 2016). These reporter genes were partially repressed in hermaphrodites relative to males, whether integrated near or far from a DCC binding site. They did not become compensated when integrated onto autosomes even when accompanied by an exceptional, high-occupancy *rex* site with multiple MEX motifs capable of binding the DCC when moved to an autosome. Third, analysis of X-chromosome transcription, combined with X-chromosome localization studies (Wheeler *et al.* 2016), provided strong evidence against a speculative model of X-chromosome dosage compensation (Sharma *et al.* 2014) in which X repression was reliant on *rex*-dependent positioning of X near the nuclear envelop in XX animals. Restricting X to a particular location within the nucleus of an XX animal does not appear to be required for its down regulation (Wheeler *et al.* 2016). Lastly, 5 of the 10 DCC subunits are homologous to subunits of the *bona fide* mitotic and meiotic condensin complexes, and four control mitotic and meiotic chromosome structure and segregation in nematodes (Csankovszki *et al.* 2009; Mets and Meyer 2009).

**Figure 9:**
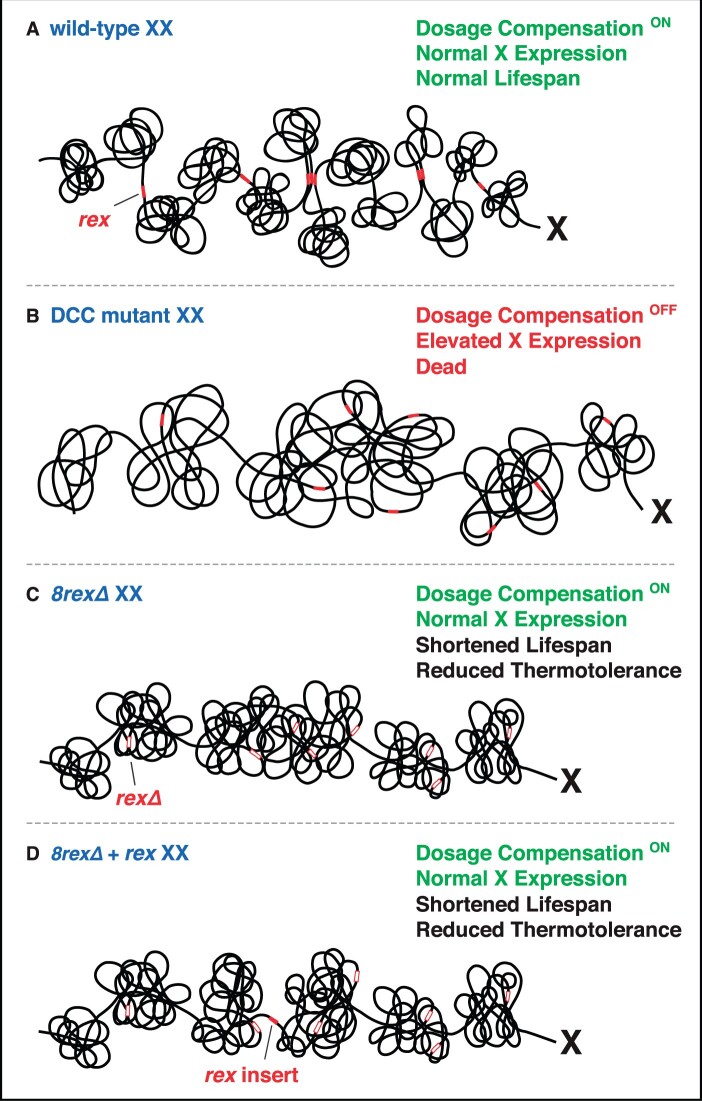
X-chromosome domain architecture established by DCC binding to *rex* sites regulates *C. elegans* lifespan but not dosage compensation. (A) DCC binding at each of eight high-occupancy *rex* sites (red rectangles) results in a TAD boundary on hermaphrodite X chromosomes. Median lifespan of wild-type XX hermaphrodites is 23 days. (B) The *sdc-2* XX mutant animals lack all DCC-dependent TAD boundaries on X, and embryos exhibit overexpression of X-linked genes and die. X-chromosome volume is expanded. (C, D) A single *rex* deletion at each boundary disrupts the boundary (C) and a single *rex* insertion (D) creates a new boundary, demonstrating that a high-occupancy *rex* site on X can be both necessary and sufficient to define a DCC-dependent boundary location. In contrast to *sdc-2* mutant embryos, *8rexΔ* mutant embryos exhibited no changes in X volume or X expression, and *8rexΔ* adults lack dosage-compensation mutant phenotypes. Hence, TAD boundaries are neither the cause nor consequence of DCC-mediated gene repression. Abrogating TAD structure did, however, reduce thermotolerance, accelerate aging, and shorten lifespan by 20% (C), implicating chromosome architecture in stress responses and aging. Inserting a *rex* site in a new location in *8rexΔ* mutants failed to suppress the reduced lifespan or reduced thermotolerance (D).

X chromosomes do indeed undergo changes in conformation during dosage compensation. Initial cytological studies measuring volumes of individual chromosomes showed that DCC binding increases compaction of X (Lau *et al.* 2014). Genome-wide chromosome conformation capture (Hi-C) studies comparing chromosome structure in wild-type *vs* DCC-defective embryos showed that the DCC remodels hermaphrodite X chromosomes into a sex-specific spatial conformation distinct from that of autosomes or male X chromosomes (Crane *et al.* 2015). The DCC creates eight self-interacting domains (∼1 megabase) resembling mammalian TADs on the dosage-compensated X chromosomes (Figure 9A). TADs on X chromosomes have stronger boundaries and more regular spacing than those on autosomes. The eight strong TAD boundaries on X coincide with the highest-affinity *rex* sites, and these boundaries become diminished or lost in mutants lacking DCC binding, thereby converting the topology of X to a conformation resembling that of autosomes (Figure 9B). The formation of DCC-dependent TAD boundaries on X also requires methylation of lysine 9 on histone H3 (H3K9me) by the methyltransferases *met-2* and *set-25* (Bian *et al.* 2020). Thus, the DCC imposes a distinct higher-order structure onto X chromosomes while regulating gene expression chromosome-wide.

### Deleting the *rex* site at each DCC-dependent TAD boundary eliminates the boundary

Mechanisms that create mammalian TAD boundaries, the biological functions of TADs, and the link to mammalian gene expression had not been well defined. In mammalian cells, architectural proteins important for establishing TADs, such as the zinc-finger protein CCCTC-binding factor (CTCF) and the SMC complex cohesin, localize at boundaries between TADs (Dixon *et al.* 2012; Nora *et al.* 2012, 2013, 2017). These architectural proteins also play roles in essential cellular processes such as chromosome segregation (Hocquet *et al.* 2018; Morales and Losada 2018), making it difficult to tease apart the functional significance of TADs from that of other vital processes by depleting these proteins (Nora *et al.* 2017; Rao *et al.* 2017; Schwarzer *et al.* 2017). Therefore, to dissect the function of TADs and the mechanism by which TAD boundaries are established, DCC-dependent TADs were analyzed by deleting DCC binding sites rather than depleting proteins essential for boundary formation (Anderson *et al.* 2019).

Individual high-occupancy *rex* sites were deleted sequentially at each of the eight DCC-dependent boundaries on the endogenous X chromosomes (*8rexΔ* XX strain) (Figure 9C) (Anderson *et al.* 2019). Deleting a single *rex* site at one of the DCC-dependent TAD boundaries on X eliminated that boundary, indicating the *rex* site was necessary to form the TAD boundary. Sequentially deleting other *rex* sites also destroyed the corresponding boundaries. Deleting all eight *rex* sites at the DCC-dependent boundaries recapitulated the disrupted TAD structure of X chromosomes in embryonic lethal *sdc-2* mutants that lack DCC binding (Figure 9C) (Anderson *et al.* 2019). In both the *8rexΔ* and *sdc-2* mutant strains, the DCC-dependent TADs were eliminated, while the weaker DCC-independent TADs on X and autosomes remained. Removing only the eight *rex* sites was sufficient to disrupt the TAD structure of X even though the DCC was bound to the numerous remaining *rex* sites across X in the *8rexΔ* XX embryos (Figure 9C). Thus, a *rex* site is necessary to define the location of each DCC-dependent TAD boundary (Anderson *et al.* 2019).

### Inserting a single high-occupancy *rex* site can be sufficient to create an ectopic TAD boundary on X, but not on autosomes

High affinity *rex* sites from existing TAD boundaries were added to new locations on X in the *8rexΔ* XX strain to determine whether a single *rex* site of this type can be sufficient to create a new TAD boundary on X (Anderson *et al.* 2019). In addition, the same *rex* sites were integrated onto autosomes in the *8rexΔ* strain to determine whether they can form boundaries outside the context of the DCC-bound X chromosome (Anderson *et al.* 2019).

A single high-occupancy *rex* site was sufficient to create a new TAD boundary on X that lacked the eight high-affinity *rex* sites, and hence all DCC-dependent TADs, or on a wild-type X with eight normal DCC-dependent TADs (Figure 9D) (Anderson *et al.* 2019). These results indicate that a high-affinity *rex* site from a TAD boundary is sufficient to create a new TAD boundary in the absence of other DCC-dependent boundaries and therefore boundary formation does not require the frequent long-range interactions that normally occur between *rex* sites at TAD boundaries. Furthermore, the native X architecture does not prevent formation of new TADs; rather the X can be further subdivided into new domains by the addition of a *rex* site. Thus, the DCC reshapes the topology of X by forming new TAD boundaries using its highest-affinity binding sites. However, not all high-occupancy *rex* sites are similar in their ability to form boundaries. A high-occupancy *rex* site from a region lacking a TAD boundary formed only a very weak boundary or no boundary at the same ectopic location that successfully formed strong boundaries from ectopic *rex* sites taken from TAD boundary locations (M. Okada and B. J. Meyer, unpublished). Special properties must exist in TAD-forming *rex* sites that have not yet been defined.

The results with autosomes were very different. Inserting three TAD-boundary *rex* sites from the center of X into the center of an autosome did not create TAD boundaries on the autosome, perhaps because the level of DCC binding at those *rex* sites on the autosome was only 20% the level of binding compared with binding at the endogenous X locations (Anderson *et al.* 2019). *rex* sites on X with the same low level of DCC binding as at those ectopic autosomal sites do not define TAD boundaries. Together these results established that the DCC forms TAD boundaries via binding to *rex* sites with high-occupancy DCC binding. A single *rex* site with high DCC occupancy can be sufficient to define a boundary and interactions with a second inserted *rex* site are not needed to strengthen the boundary. Analysis of changes in chromosome structure during dosage compensation was the first study to identify the machinery and DNA sites that create chromosome-wide TAD structure and then to use specific *cis-*acting mutations to disrupt it.

**Figure 10:**
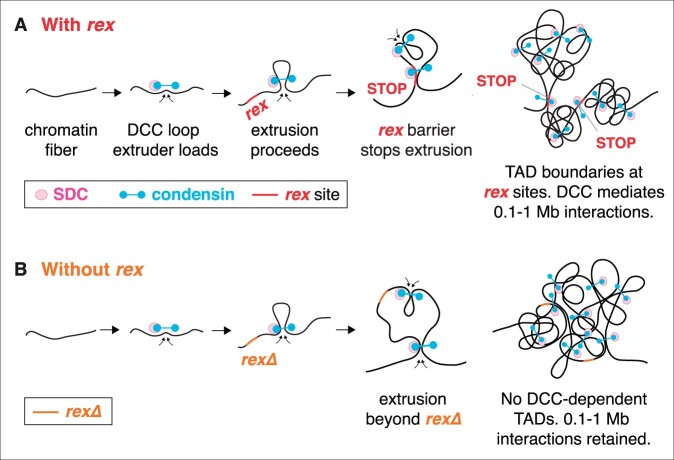
Loop extrusion model for TAD formation by the DCC. (A) The popular model about TAD boundary formation called loop extrusion is supported by available data for the induction of X-chromosome structure by the DCC. In this model, the DCC condensin (blue) loads onto chromatin with SDC proteins (magenta) and extrudes loops of increasing size until the extrusion is halted by binding to a high-occupancy *rex* site with multiple X-enriched motifs (red). Because DCC-mediated loops do not cross high-occupancy *rex* sites, the *rex* sites define the locations of TAD boundaries. The SDC loading factors could travel with condensin subunits from loading sites on X to the highest-affinity *rex* sites, where they bind stably and block extrusion. Alternatively, condensin alone could bind at low levels to some X sites without SDC proteins and extrude loops until encountering SDC proteins bound at a *rex* site. Only boundary *rex* sites are drawn, even though numerous *rex* sites with a range of DCC binding affinities act as loading sites and confer X specificity. (B) When high-occupancy *rex* sites are deleted (orange), TAD boundaries are lost, but other DCC-mediated DNA interactions remain, most notably those in the 0.1–1 Mb length scale. The *8rexΔ* X maintains the same level of compaction as the wild-type X.

Instead of favoring a model in which specific DCC-mediated interactions promote TAD boundary formation, the current evidence supports a loop extrusion model for boundary formation and chromosome compaction (Figure 10) (Anderson *et al.* 2019; Rowley *et al.* 2020). In this model, a protein complex extrudes a chromatin loop of increasing size until it reaches a barrier element that blocks the progression of extrusion (Fudenberg *et al.* 2016; Nuebler *et al.* 2018). Different DCC components would function to extrude loops and block extrusion, analogous to the roles played in mammals by the SMC complex cohesin, which extrudes loops, and the zinc finger DNA-binding protein called CTCF, which acts as a barrier to loop extrusion when cohesin encounters CTCF at a high-occupancy binding site. DCC condensin subunits would extrude loops, and noncondensin subunits such as SDC-2 would halt extrusion when bound at one of the highest-occupancy *rex* sites that drive boundary formation (Anderson *et al.* 2019) (Figure 10).

### X-chromosome TAD domain structure does not regulate dosage compensation

To determine if TADs regulate dosage compensation, *8rexΔ* XX worms were examined for evidence of canonical dosage compensation defects. Complete disruption of dosage compensation causes XX-specific lethality, and weak disruption causes dumpy (Dpy) and egg-laying defective (Egl) phenotypes (Plenefisch *et al.* 1989). In addition, mutations that disrupt dosage compensation rescue the XO-specific lethality caused by mutations in *xol-1* (Miller *et al.* 1988; Rhind *et al.* 1995). The *8rexΔ* XX worms lacked dosage-compensation mutant phenotypes. They had normal brood sizes, and 100% of animals were viable and neither Dpy nor Egl. In addition, the *8rexΔ* mutations failed to restore viability to *xol-1* mutant XO males (Anderson *et al.* 2019).

A more direct and sensitive monitor of defects in dosage compensation was achieved by comparing genome-wide gene expression using RNA-seq across three different genotypes of embryos: wild-type XX embryos, DCC-defective XX mutant embryos lacking DCC binding on X, and *8rexΔ* XX embryos with altered X topology but persistent DCC binding on X (Anderson *et al.* 2019). While median X expression was elevated 1.5-fold in DCC mutant *vs* wild-type embryos, expression of X genes was not elevated in *8rexΔ* XX embryos (Figure 9, A, B, and C). Moreover, for DCC-defective XX embryos, no correlation was evident between any changes in gene expression and distance to the location of a DCC-dependent boundary found in wild-type embryos. In both *8rexΔ* and DCC mutant embryos, X-chromosome TAD boundaries were lost, but X-linked gene expression was elevated only in the DCC mutant. Therefore, DCC-dependent TADs are neither the cause nor consequence of transcriptional repression; the changes in chromosome domain architecture and gene expression result from two separate DCC roles (Anderson *et al.* 2019).

### Eliminating DCC-dependent TADs on X reduces thermotolerance, accelerates aging, and shortens lifespan

Although the disruption of X structure in the *8rexΔ* XX strain did not cause statistically significant changes in embryonic gene expression under normal growth conditions, it did adversely affect the ability of adults to tolerate induced proteotoxic stress (Anderson *et al.* 2019). The presence of unfolded proteins in worms exposed to proteotoxic stress triggers activation of genes needed to refold proteins and restore homeostasis (Higuchi-Sanabria *et al.* 2018). Lack of response causes death. The viability of *8rexΔ* XX adults was significantly reduced by the presence of unfolded proteins caused by heat stress (37°C for 7 h) (Anderson *et al.* 2019) (Figure 9C). However, *8rexΔ* XX animals were not more sensitive to paraquat-induced mitochondrial stress, which causes accumulation of reactive oxygen species or to tunicamycin-induced endoplasmic reticulum (ER) stress, which causes accumulation of unfolded glycoproteins. Thus, removing DCC-dependent TADs specifically impairs thermotolerance but does not compromise responses to all forms of proteotoxic stress (Anderson *et al.* 2019).

The *8rexΔ* XX hermaphrodites also exhibited a 20% reduction in lifespan, and inserting a *rex* site at a new location on X did not restore the lifespan (Figure 8, A, C, and D) (Anderson *et al.* 2019). The decrease in lifespan appears to be the consequence of disrupting the hermaphrodite-specific and DCC-dependent functions of *rex* sites, because the lifespan of males was not affected by the *rex* deletions. Several changes in behavior, including a premature decline in both speed and distance of backward movement after reaching adulthood normally, indicated that the *8rexΔ* XX hermaphrodites died prematurely from accelerated aging rather than from general “sickness” during development (Anderson *et al.* 2019). Thus, although *rex* deletions that abrogate TAD structure in hermaphrodites do not affect DCC-regulated gene expression during embryogenesis, they increase thermosensitivity, accelerate aging, and shorten lifespan during adulthood, implying a role for chromosome architecture in regulating stress responses and aging (Anderson *et al.* 2019).

### TAD-independent DCC-mediated architecture

The complete disruption of DCC-dependent TAD boundaries on *8rexΔ* chromosomes led to the discovery of an additional level of DCC-dependent architecture on X that has the potential to facilitate gene repression. In the absence of TADs, DCC-dependent high-frequency interactions persisted between loci spanning 0.1 and 1 Mb (Anderson *et al.* 2019) (Figure 10). These DCC-dependent 0.1–1 Mb interactions have the potential to contribute to X compaction and long-range gene repression by creating X segments with environments unfavorable for RNA polymerase II recruitment. The DCC does reduce the volume of X in addition to creating TADs, but eliminating TADS on *8rexΔ* X chromosomes did not change the X volume (Figure 9, A and C), revealing another TAD-independent DCC-mediated change in X architecture. In contrast, an *sdc-2* mutation did increase X volume in addition to disrupting the 0.1–1 Mb interactions (Figure 9B). Although TAD boundaries do not mediate X compaction, the DCC-dependent 0.1–1 Mb interactions might (Anderson *et al.* 2019).

### The DCC creates local negative supercoils at *rex* sites, but does not create large domains of supercoiling along X

The predominant form of DNA in the cell is a double-stranded, right-handed helix with 10.4 bp per helical turn, but biological process such as transcription can either overwind or underwind DNA, thereby creating DNA supercoils (Kouzine and Levens 2007). Supercoils have been proposed to regulate higher-order chromosome structure and chromosome-wide gene expression (Racko *et al.* 2019). While it is well accepted that prokaryotic chromosomes are organized into supercoiled topological domains (Postow *et al.* 2004), the role of supercoils in eukaryotic structure has been controversial. Using condensin-driven dosage compensation as a model, the relationship between 3D chromosome topology, condensin, and supercoiling were investigated *in vivo* (Krassovsky *et al.* 2021). Initial experiments demonstrated that X-chromosome repression is not achieved by regulating supercoiling at TSS of X-linked genes (Krassovsky *et al.* 2021). Further experiments showed that many high-occupancy *rex* sites have local DCC-dependent negative supercoils: The supercoils were observed in wild-type animals but not in DCC mutants. The level of supercoiling correlated positively with the strength of DCC binding. Even though the supercoils occur at the very *rex* sites that trigger formation of TAD boundaries, the supercoils do not propagate beyond 500 bp, a distance less than the size of a TAD or even a TAD boundary itself (Krassovsky *et al.* 2021). Hence, the limited DCC-dependent supercoiling at *rex* sites cannot change DNA interaction frequencies on the mega-base scale needed to create a TAD. Supercoiling domains of 0.1–1 Mb were also not found, indicating that the DCC-dependent 0.1–1 Mb interactions that might contribute to the regulation of gene expression are not caused by supercoiling (Krassovsky *et al.* 2021).

### The DPY-21 histone H4K20 demethylase enriches H4K20me1 on X chromosomes and controls the topology and repression of X

**Figure 11:**
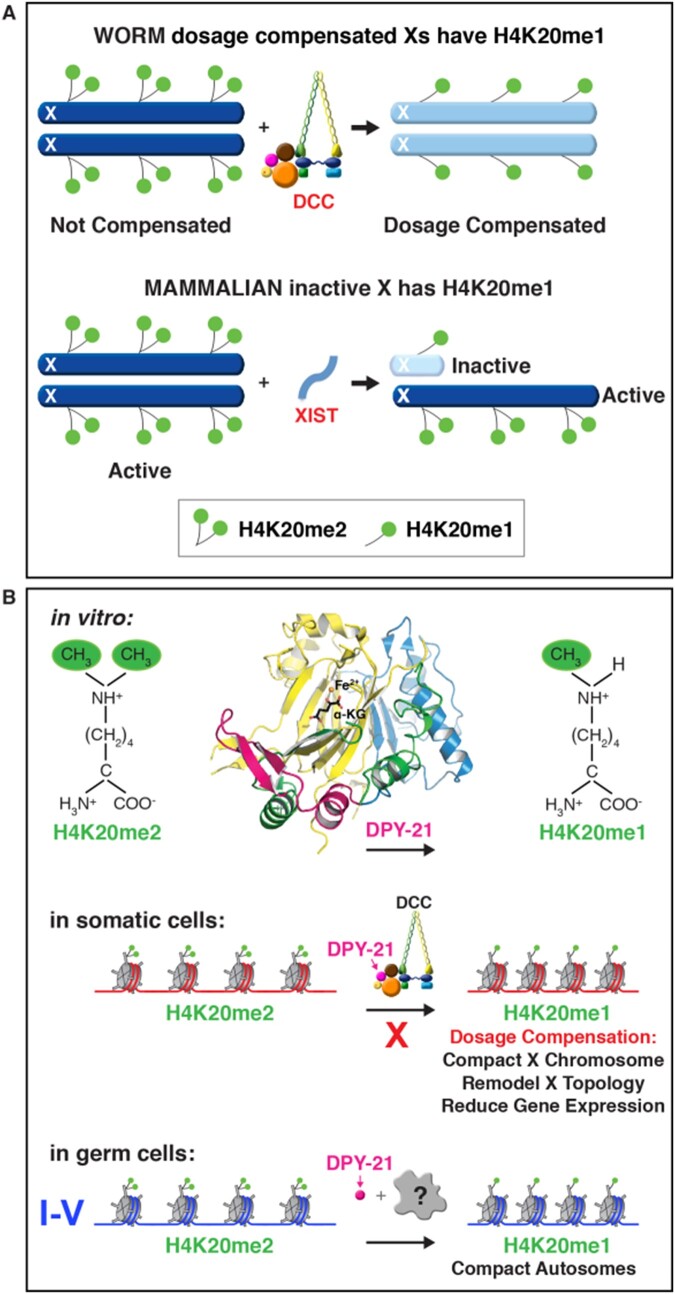
Control of X-chromosome histone modification, topology, and repression by a histone H4K20 demethylase DCC subunit that catalyzes formation of H4K20me1. (A) During the establishment and maintenance of dosage compensation, the DCC enriches the histone modification H4K20me1 on both hermaphrodite X chromosomes. H4K20me1 is also enriched on the inactive X chromosome of female mammals, revealing a common feature of diverse dosage compensation strategies. (B) The DPY-21 H4K20me2 histone demethylase regulates 3D X-chromosome structure and gene expression by catalyzing enrichment of H4K20me1. The 1.8 Å crystal structure of DPY-21 and biochemical assays *in vitro* identified a novel, highly conserved H4K20me2 JmjC demethylase subfamily that converts H4K20me2 to H4K20me1 in an Fe^2+^ and α-ketoglutarate-dependent manner. In somatic cells, DPY-21 binds to X chromosomes via the DCC and enriches H4K20me1 to repress gene expression. The H4K20me1 enrichment controls the higher-order structure of X chromosomes by facilitating compaction and TAD formation. In germ cells, DPY-21 enriches HK20me1 on autosomes, but not X, in a DCC-independent manner to promote chromosome compaction.

During the establishment and maintenance of dosage compensation, the chromatin modification H4K20me1 is enriched on hermaphrodite X chromosomes in a DCC-dependent manner (Liu *et al.* 2011; Vielle *et al.* 2012; Wells *et al.* 2012; Kramer *et al.* 2015; Brejc *et al.* 2017) (Figure 11A). X chromosomes of males and DCC-defective hermaphrodites lack this monomethylation of lysine 20 on histone H4. H4K20me1 is also enriched on the inactive X chromosome of female mammals, revealing a common feature of diverse dosage compensation strategies (Figure 11A) (Kohlmaier *et al.* 2004). In general, the role of H4K20me1 in gene regulation was a puzzle due to its context-dependent contribution to both gene activation and gene repression (Beck *et al.* 2012). Moreover, the impact of histone modifications on higher-order chromosome structure beyond chromatin fiber compaction was not well understood. Although H4K20 methylation had been implicated in many nuclear functions, including DNA replication and repair, gene regulation, mitotic chromosome condensation, and cell-cycle control, the mechanisms that regulate different HK20me states (H4K20me1/me2/me3) and that transduce these states into properly executed nuclear functions had not been well understood (Beck *et al.* 2012; Jorgensen *et al.* 2013; van Nuland and Gozani 2016). Analyzing the causes and consequences of H4K20me1 modification during dosage compensation provided an excellent model for such investigations.

In principle, H4K20me1 enrichment on *C. elegans* X chromosomes could occur by activating the methyltransferase (SET-1) that converts H4K20me0 to H4K20me1, by blocking the methyltransferase (SET-4) that converts H4K20me1 to H4K20me2/me3, by inhibiting the demethylase (JmJD-1.1/1.2) that converts H4K20me1 to H4K20me0, or by activating an unknown demethylase that converts H4K20me2 to H4K20me1. Although H4K20me2 is the predominant form of H4K20 in eukaryotic cells (Pesavento *et al.* 2008), only a neuron-specific H4K20me2 demethylase had been reported for any organisms (Wang *et al.* 2015). The first published models for H4K20me1 enrichment on *C. elegans* X chromosomes featured the inhibition of SET-4 as the likely mechanism (Vielle *et al.* 2012; Wells *et al.* 2012). Instead, a DCC subunit itself was found to act as an H4K20me2 demethylase to enrich H4K20me1 on X (Brejc *et al.* 2017).

Although amino acid sequence analysis failed to identify a demethylase domain in any of the DCC subunits, structure prediction programs suggested homology between the carboxyl-terminal domain of DPY-21 and Jumonji (JmjC) domain-containing lysine demethylases. JmjC demethylases are Fe^2+^ and α-ketoglutarate (α-KG)-dependent dioxygenases that demethylate lysines in histone and nonhistone proteins. A 1.8 Å crystal structure of the putative DPY-21 JmjC domain and biochemical assays of both the purified DPY-21 domain and the homologous C-terminal domain of mouse protein ROSBIN (round spermatid basic protein) revealed that DPY-21 defines a new subfamily of JmjC histone demethylases that convert H4K20me2 to H4K20me1 *in vitro* and is widely conserved from worms to mammals (Figure 11B) (Brejc *et al.* 2017). Amino acid substitutions of alanine for DPY-21 residues H1452 and D1454, predicted to coordinate α-KG and chelate Fe^2+^, caused loss of H4K20me2 demethylase activity *in vitro*, demonstrating a Fe^2+^-dependent and α-KG-dependent mechanism for histone H4K20me2 demethylation (Brejc *et al.* 2017).

Analysis of DPY-21 JmjC demethylase activity *in vivo* revealed that DPY-21 acts in a cell-cycle-dependent manner to enrich H4K20me1 on hermaphrodite X chromosomes of somatic cells (Figure 11B) (Brejc *et al.* 2017). H4K20me1 is specifically enriched on X only during interphase. During mitosis, H4K20me1 levels are uniformly elevated on all chromosomes in a DPY-21-independent manner. H4K20me1 enrichment is not evident on interphase X chromosomes before the 200-cell stage of embryogenesis, long after initial recruitment of SDC-2 and other DCC subunits to X (30- to 40-cell stage), implying a more prominent role in maintenance of dosage compensation rather than initiation. In contrast to all other DCC subunits, DPY-21’s association with X is precisely coincident with the timing of H4K20me1 enrichment on X, and DPY-21 does not associate with mitotic chromosomes. Furthermore, enrichment of H4K20me1 on X chromosomes is absent in *dpy-21(JmjC)* mutants lacking H4K20me2 demethylase activity, indicating that DPY-21 is responsible for the enrichment of H4K20me1 on X *in vivo*.

Inactivation of DPY-21 demethylase activity *in vivo* also revealed that H4K20me1 enrichment is essential for repression of X-chromosome gene expression (Bian *et al.* 2017; Brejc *et al.* 2017). Direct measurements of mRNA levels showed that X-linked gene expression is elevated in *dpy-21(JmjC)* mutant hermaphrodites, indicating disruption of dosage compensation. Also, *dpy-21(JmjC)* mutations prevented the death of *xol-1* XO males caused by DCC binding to the single X and the consequent reduction of X-chromosome gene expression.

Inactivation of demethylase activity also reduced X compaction in somatic cells by causing a 30% increase in X volume. In addition, the inactivation disrupted X conformation by diminishing DCC-dependent TAD formation. The strength of all DCC-dependent TAD boundaries on X, but not any DCC-independent boundaries, was reduced significantly. These results indicate DPY-21 JmjC activity is important for both the compaction of X and the DCC-driven remodeling of X topology (Figure 11B) (Brejc *et al.* 2017).

DPY-21 also binds to autosomes (but not X chromosomes) of meiotic germ cells in a DCC-independent matter to enrich H4K20me1 and compact chromosomes (Figure 11B) (Brejc *et al.* 2017). Inactivation of DPY-21 causes a 20% increase in lengths of autosomal axes. DPY-21 lacks obvious DNA and chromatin-binding domains to confer target specificity, allowing the demethylase activity to be harnessed during development for distinct biological functions by targeting it to diverse genomic locations. In both somatic and germ cells, H4K20me1 modulates 3D chromosome topology, showing a direct link between chromatin modification and higher-order chromosome structure (Brejc *et al.* 2017). DPY-21’s roles in chromatin modification and chromosome topology further illustrate how the dosage compensation process evolved by co-opting conserved machinery used in other biological processes for the new task of fine-tuning X-chromosome gene expression.

Enrichment of H4K20me1 on the inactive X chromosome of XX female mammals underscores the relevance of *C. elegans* H4K20me1 studies for mammalian development (Kohlmaier *et al.* 2004). Knockout of the mammalian H4K20me1 methyl transferase causes loss of H4K20me1 enrichment on the inactive X and the consequent decondensation of X (Oda *et al.* 2009). H4K20me1 enrichment is dependent on the long noncoding RNA *Xist*, the trigger of mammalian X inactivation, but partial-loss-of-function *Xist* mutations that prevent H4K20me1 enrichment on X can nonetheless permit X inactivation with lower efficiency (Tjalsma *et al.* 2021). These results suggest that the function of H4K20me1 on X is to facilitate chromatin compaction that is a characteristic of facultative heterochromatin on the inactive X rather than to initiate early gene silencing on X (Tjalsma *et al.* 2021). Analysis of H4K20me1 in worms and mammals offers new directions for unraveling the interplay between chromatin modification and chromosome structure.

### Overview and future directions

Evolution of the dosage compensation process in *C. elegans* required the recruitment of proteins from ancient condensin complexes that induce chromosome restructuring and segregation to the new role of regulating chromosome-wide gene expression. Condensin proteins co-opted for dosage compensation also retained their original roles in chromosome segregation by maintaining their participation in other nematode condensin complexes. This co-option demonstrates that reshuffling of homologous interchangeable molecular parts can create independent machines with similar architecture but distinct cellular localization and biological functions.

Future research will determine how different nematode DCC proteins nucleate and spread on X chromosomes and how chromatin structure responds to and/or regulates the DCC nucleation and spreading process. Single-molecule imaging of individual DCC subunits in live embryos will reveal the dynamic nature of DCC assembly and spreading and the degree to which DCC condensin subunits function in an independent subcomplex to alter chromosome topology. This information will help elucidate the mechanism by which DCC binding on X regulates RNA polymerase recruitment and transcription attenuation in hermaphrodites. Precision mapping of cell-specific X-chromatin compaction at local and global scales in individual embryonic cells combined with single-molecule imaging of DCC proteins and RNA polymerase and high-resolution analysis of the cellular transcriptome in the same cells will yield an unprecedented depth of understanding not just about the mechanisms of dosage compensation, but also the degree to which protein complex structures inferred from biochemical analysis correspond to the dynamic structures of complexes in living cells.
